# Robust Significance Analysis of Microarrays by Minimum *β*-Divergence Method

**DOI:** 10.1155/2017/5310198

**Published:** 2017-07-27

**Authors:** Md. Shahjaman, Nishith Kumar, Md. Manir Hossain Mollah, Md. Shakil Ahmed, Anjuman Ara Begum, S. M. Shahinul Islam, Md. Nurul Haque Mollah

**Affiliations:** ^1^Bioinformatics Lab, Department of Statistics, University of Rajshahi, Rajshahi 6205, Bangladesh; ^2^Department of Statistics, Begum Rokeya University, Rangpur, Rangpur 5400, Bangladesh; ^3^Department of Statistics, Bangabandhu Sheikh Mujibur Rahman Science and Technology University, Gopalganj, Bangladesh; ^4^Department of Biostatistics, BUHS, Dhaka, Bangladesh; ^5^Institute of Biological Science (IBSc), University of Rajshahi, Rajshahi 6205, Bangladesh

## Abstract

Identification of differentially expressed (DE) genes with two or more conditions is an important task for discovery of few biomarker genes. Significance Analysis of Microarrays (SAM) is a popular statistical approach for identification of DE genes for both small- and large-sample cases. However, it is sensitive to outlying gene expressions and produces low power in presence of outliers. Therefore, in this paper, an attempt is made to robustify the SAM approach using the minimum *β*-divergence estimators instead of the maximum likelihood estimators of the parameters. We demonstrated the performance of the proposed method in a comparison of some other popular statistical methods such as ANOVA, SAM, LIMMA, KW, EBarrays, GaGa, and BRIDGE using both simulated and real gene expression datasets. We observe that all methods show good and almost equal performance in absence of outliers for the large-sample cases, while in the small-sample cases only three methods (SAM, LIMMA, and proposed) show almost equal and better performance than others with two or more conditions. However, in the presence of outliers, on an average, only the proposed method performs better than others for both small- and large-sample cases with each condition.

## 1. Introduction

Microarray experiments are usually conducted with expressions of huge number of genes (*G*) and a small number of experimental samples (*n*). This unique data structure has been discovered as a completely new promising area for the researchers. At the same time, it provides a challenge to the researchers because of high dimensionality and its complexity with small sample size. Among this huge number of genes, discovery of few biomarker genes those are differentially expressed (DE) between two or more experimental conditions with multiple patterns is one of the main objectives of this experiments. These biomarker genes are important in the diagnosis of different types and subtypes of diseases for patient prognosis and treatment [1–3]. Nowadays, researchers are also interested in exploring the gene coexpression network or interaction of DE genes to predict the hub genes that are associated with different types and subtypes of cancer [4]. The most commonly used statistical tests for the discovery of DE genes between two or more conditions are* t*-test or ANOVA (*F*-test). However, both testing procedures sometimes produce misleading results to discover few biomarker genes, because both of them suffer from small-sample sizes and normality assumptions, and they do not share the information of all genes [5]. Therefore, a gene-specific* t*-statistic or* F*-statistic becomes large even for low differential expressions of genes between two or more conditions. Thus, the false discovery rate (FDR) may increase. Tusher et al. [6] introduced a popular statistical technique to detect the DE genes by assimilating a set of gene-specific* t*-tests. This approach is known as Significance Analysis of Microarrays (SAM). It controls the FDR by sharing information of all genes. It does not suffer from the small-sample sizes and normality assumptions. There is a close relation between SAM and FDR-based multiple testing correction approach of Benjamini and Hochberg [7]. Because of low expense and advancement of microarray technology, SAM has been extensively used in gene expression data analysis. For example, Li et al. [8] performed an integrative data analysis to identify the breast cancer subtype-specific biomarkers by combining copy number aberrations and miRNA-mRNA dual expression profiling data. Tarhini et al. [9] identified some immune-related genes that are associated with neoadjuvant ipilimumab clinical benefit. Wu et al. [10] detected some potential biomarkers for the diagnosis and prediction of preeclampsia. Ren et al. [11] identified some subtype-specific novel biomarkers using colon cancer gene expression data. However, SAM is very sensitive to outliers. It produces larger FDR and lower power in presence of outliers. Therefore, in this paper, an attempt is made to robustify the SAM [6] approach using the minimum *β*-divergence method [12]. Then we investigate the performance of the proposed method in a comparison of traditional SAM approach as well as some other popular methods such as ANOVA [13, 14], LIMMA [15], Kruskal-Wallis (KW) [16], empirical Bayes (EB) [17–19], Gama-Gama model (GaGa) [20], and Bridge [21] using both simulated and real gene expression datasets. The paper is organized as follows: Section 2 contains the formulation of traditional SAM algorithm and proposed robust SAM algorithm with detailed description. Simulation and real microarray data analysis are carried out in Section 3. Finally, we end up with a conclusion.

## 2. Methods

Let *x*_*ij*_ be the *i*th gene expression for the *j*th samples (*i* = 1,2,…, *G*; *j* = 1,2,…, *n*_*k*_; *k* = 1,2). Also let *μ*_*ik*_ denote the mean of the *i*th gene for* k*th condition. Then we would like to test the hypothesis H_0_: *μ*_*i*1_ = *μ*_*i*2_ versus H_1_: *μ*_*i*1_ ≠ *μ*_*i*2_ which implies that H_0_: *μ*_*i*1_ − *μ*_*i*2_ = 0 versus H_0_ is not true. A gene is said to be equally expressed (EE) if H_0_ is accepted; otherwise it is DE. If ${\hat{\mu }}_{ik}$ denotes the sample mean of *i*th gene for *k*th condition and *s*_*i*_^2^ denotes the pooled within-class sample variance then the formula of two-sample* t*-test to test the above null-hypothesis is as follows:

(1) $$\begin{array}{c} t_{i}=\frac{r_{i}}{s_{i}}, \end{array}$$

where

(2) ri=μ^i1−μ^i2,si=an1−1σ^i12+n2−1σ^i221/2.

Here, *a* = 1/∑_*k*_(*n*_*k*_ − 1) · (∑_*k*_(1/*n*_*k*_)),

(3) μ^ik=∑jxijnkσ^ik2=1nk−1∑k∑jxij−μ^ik2.

The* t*-statistic given in (1) follows the* t*-distribution with (*n*_1_ + *n*_2_ − 2) degrees of freedom. As early mentioned this test statistic increases the FDR for small-sample cases. To overcome this problem Tusher et al. [6] proposed a modification of the* t*-statistic by adding a constant *s*_0_ to the denominator, which is known as the test statistic of Significance Analysis of Microarrays (SAM) algorithm. This statistic is defined as follows:

(4) $$\begin{array}{c} t_{i}^{\text{SAM}}=\frac{r_{i}}{s_{i}+s_{0}}, \end{array}$$

where *s*_0_ is the percentile of the distribution of *s*_*i*_. For *k* > 2 conditions, the modified* t*-statistic in (4) is defined in terms of Fisher's linear discriminant, assuming *n* samples consist of *m* nonoverlapping subsets, such that the response parameter *y*_*j*_ ∈ {1,2,…, *m*}, *C*_*k*_ = {*j*:  *y*_*j*_ = *k*}, and *n*_*k*_ is the number of expressions in *C*_*k*_. Then the scores *r*_*i*_ and standard deviation *s*_*i*_ in (4) are replaced by the following two equations, respectively:

(5) ri=∑j∈Cknk∏nk∑k=1mnkμ^ik−μ^i21/2si=an1−1σ^i12+n2−1σ^i22+⋯+nk−1σ^ik21/2,

where ${\hat{\mu }}_{i}=\sum_{k}n_{k}{\hat{\mu }}_{ik}/\sum_{k}n_{k}$. For the details about SAM procedure visit http://statweb.stanford.edu/~tibs/SAM/.

However, the test statistic given in (4) produces misleading results in presence of outliers, since ${\hat{\mu }}_{ik}$ and ${\hat{\sigma }}_{ik}^{2}$ in (2) and (5) are sensitive to outliers. Therefore, in this paper, an attempt is made to robustify the test statistic, *t*_*i*_^SAM^, in (4) by minimum *β*-divergence method. The minimum *β*-divergence estimators ${\hat{\theta }}_{ik,\beta }=\left({\hat{\mu }}_{ik,\beta },{\hat{\sigma }}_{ik,\beta }^{2}\right)$ of the parameters ***θ***_*ik*_ = (*μ*_*ik*_, *σ*_*ik*_^2^) are computed iteratively as follows:

(6) μik,t+1=δ∑j∈Ckφβxij ∣ θik,txij,σik,t+12=β+1δ∑j∈Ckφβxij ∣ θik,txij−μik,t2,

where *δ* = 1/∑_*j*∈*C*_*k*__*φ*_*β*_(*x*_*ij*_∣***θ***_*ik*,*t*_). Here

(7) $$\begin{array}{c} \varphi_{\beta }\left(\;x_{ij}\;|\;\theta_{ik,t}\;\right)=\exp\left\{\;-\frac{\beta }{2\sigma_{ik}^{2}}{\left(\;x_{ij}-\mu_{ik}\;\right)}^{2}\;\right\}, \end{array}$$

which is known as *β*-weight function. This weight function was first introduced in [12] for the robustification of prewhitening procedure to improve the performance of independent component analysis (ICA) algorithms for blind source separation (BSS). It was generalized in [22] for the robust extraction of local principal components. Then it was used in [23] for the robustification of empirical Bayes approach [18] to identify DE genes between two conditions. It was also used in [24] to improve one-way ANOVA for the robust and efficient estimation of DE genes with multiple patterns. Note that if *β* = 0, the minimum *β*-divergence estimators ${\hat{\theta }}_{ik,\beta }=\left({\hat{\mu }}_{ik,\beta },{\hat{\sigma }}_{ik,\beta }^{2}\right)$ reduce to classical maximum likelihood estimators (MLEs) ${\hat{\theta }}_{ik}=\left({\hat{\mu }}_{ik},{\hat{\sigma }}_{ik}^{2}\right)$. Since, in absence of outliers, the MLEs of Gaussian distribution are consistent and asymptotically efficient [25], therefore, in this paper, the MLEs are used in absence of outliers and in presence of outliers, the minimum *β*-divergence estimators are used for the estimation of ***θ***_*ik*_ in SAM approach. We can apply our proposed SAM approach in two ways. One way is to select the *β* using the cross validation (CV) which was discussed in detail in [12, 22]. The CV approach produces *β* = 0 in absence of outliers, while *β* > 0 in presence of outliers. The minimum *β*-divergence estimators in (6) with *β* = 0 are equivalent to MLEs in (3) as mentioned previously. Thus the minimum *β*-divergence estimators with an appropriate *β* selection by CV produce both robust and efficient estimates for the parameters. However, in our current problem, it would be time-consuming, since CV approach needs to be applied for each gene of the entire genome in each condition separately to select the appropriate *β*. To overcome this problem, in this paper we consider outlier detection approach based on *β*-weight function with a fixed *β* > 0. The value of *β*-weight function lies between 0 and 1. It produces larger weights with the usual gene expressions and smaller weights with the unusual/outlying gene expressions for a wide range of *β* > 0 [12, 22]. By assigning low weights to outliers, the estimators become robust. The larger *β* increases the robustness of estimators but decreases the efficiency, while the smaller *β* increases the efficiency but decreases the robustness. Thus the value of *β* controls the balance between the robustness and efficiency of the estimators. Therefore, in this paper, we fix *β* = 0.2 for outlier detection using *β*-weight function which was also used in [24]. A value (*x*_*ij*_) of gene expression is classified as usual or unusual based on this *β*-weight function as follows:

(8) $$\begin{array}{c} \varphi_{\beta }\left(\;x_{ij}\;|\;{\hat{\theta }}_{ik,\beta }\;\right)=\left\{\begin{array}{cc} \leq \lambda_{i}, & \text{If }j\text{th expression for }i\text{th gene is contaminated by outlier in the }k\text{th condition}\\ >\lambda_{i}, & \text{otherwise,} \end{array}\right. \end{array}$$

where we fix the cutoff value *λ*_*i*_ = min⁡(0.2, *λ*_*i*0_), since *β*-weights lie between 0 and 1 and smaller weights occur with unusual/outlying gene expressions. Here *λ*_*i*0_ is calculated by the following equation:

(9) λi0=min⁡φβxij ∣ θ^ik,β+γmax⁡φβxij ∣ θ^ik,β−min⁡φβxij ∣ θ^ik,β∀i,j,k,

which was also used in [22]. Here, *γ* is a smaller quantity; in our analysis we consider *γ* = 0.1. After convergence of (6), we obtained the robust estimates ${\hat{\theta }}_{ik,\beta }=\left({\hat{\mu }}_{ik,\beta },{\hat{\sigma }}_{ik,\beta }^{2}\right)$ of the parameters ***θ***_*ik*_ = (*μ*_*ik*_, *σ*_*ik*_^2^). Then we combine the MLEs and minimum *β*-divergence estimators to estimate the parameters ***θ***_*ik*_ = (*μ*_*ik*_, *σ*_*ik*_^2^) as follows:

(10) $$\begin{array}{c} {\hat{\theta }}_{ik}^{*}=\left\{\begin{array}{cc} {\hat{\theta }}_{ik,\beta }, & \text{If }i\text{th gene expression is contaminated by outlier in the }k\text{th condition}\\ {\hat{\theta }}_{ik}, & \text{otherwise.} \end{array}\right. \end{array}$$

## 3. Results and Discussion

To demonstrate the performance of the proposed method in a comparison of other popular methods (ANOVA, SAM, LIMMA, KW, EB, GAGA, and BRIDGE), we used both simulated and real microarray gene expression datasets. We used five R packages of other methods such as samr, limma, EBarrays, gaga, and bridge. The performance measures AUC and pAUC were computed for each of the methods using ROC package. All R packages are available in the comprehensive R archive network (cran) or bioconductor.

### 3.1. Performance Evaluation

In order to investigate the performance of the proposed method in a comparison of some other popular methods for binary class prediction such as DE or EE, we use different performance measures including the receiving operating characteristic (ROC) curve, area under the ROC curve (AUC), and partial AUC (pAUC) derived through the confusion matrix as shown in Table 1.

We compute different performance index based on the confusion matrix as follows:

  True positive rate (TPR) = *n*TP/(*n*TP + *n*FN), true negative rate (TNR) = *n*TN/(*n*TN + *n*FP).
  False positive rate (FPR) = *n*FP/(*n*FP + *n*TN), false negative rate (FNR) = *n*FN/(*n*FN + *n*TP).
  False discovery rate (FDR) = *n*FP/(*n*TP + *n*FP), misclassification error rate (MER) = (*n*FP + *n*FN)/(*n*TP + *n*TN + *n*FP + *n*FN).
  Power = 1 − FNR, where *n*TP denotes the number of true positives and so on.

A method is said to be good performer if it produces higher values of TPR, TNR, AUC, pAUC, and power and smaller values of FPR, FNR, FDR, and MER.

### 3.2. Data Generating Model

We used the following statistical model to generate simulated data with known characteristics:

(11) xij=μik+ϵij;i=1,2,…,G;  j=1,2,…,nk;  k=1,2,…,m,

where *x*_*ij*_ is the *i*th gene expression for the* j*th samples, *μ*_*ik*_ is the mean of all expressions of *i*th gene in the *k*th condition, and *ϵ*_*ij*_ is the random error term which follows *N*(0, *σ*^2^). The outlying datasets were generated by replacing at most 5% of the expression values from *n* = *n*_1_ + *n*_2_ + ⋯+*n*_*k*_ samples by outliers using

(12) xij∗=z+2∗max⁡xij;  i=1,2,…,G;  j=1,2,…,nk;  k=1,2,…,m,

where *z* ∈ (5,10) is an arbitrarily fixed value.

#### 3.2.1. Simulation Study 1

**Figure 1:**
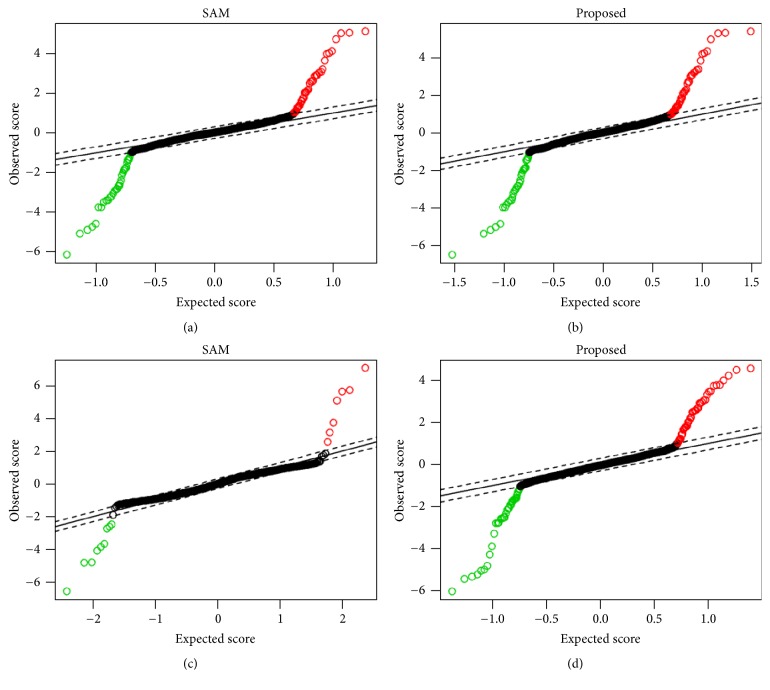
Performance evaluation using* Q-Q* plot for detection of DE genes by SAM and proposed method. (a-b) In absence of outliers. (c-d) In presence of outliers.

To investigate the performance of the proposed robust SAM in a comparison with the traditional SAM approach, we generated a dataset using (11) with *G* = 1,000 genes, and *n* = 20 samples, 10 each in condition 1 and condition 2. There are 100 DE genes in this dataset (50 upregulated and 50 downregulated). Then we employed SAM and proposed method in this dataset to determine the DE genes. Figures 1(a) and 1(b) represent the* Q-Q* plots for this dataset using SAM and proposed method. In this figure, the number of genes above the band in the upper right (red color) and below the band in the bottom left (green color) indicates the number of upregulated and downregulated DE genes, respectively. We observed that both SAM and our proposed method identified 88 true DE genes with Δ = 0.2 considering 12 false positives on an average. To evaluate the performance of these two methods in presence of outliers, we generate the outlying dataset using (12). We consider one outlier in each of 10% genes. Then we employed these methods in the outlying dataset to identify the DE genes. Figures 1(c) and 1(d) show the* Q-Q* plots using this outlying dataset. We can clearly see from Figure 1(c) that there are only 19 true DE genes identified by the SAM with Δ = 0.1 considering 42 false positives on an average, whereas in Figure 1(d) the proposed method identified 86 true DE genes with Δ = 0.2 considering 14 false positives on an average. The plots of smallest *β*-weight for each of 1000 genes are displayed in Figures S1(a) and S1(b) in the supporting file (see Supplementary Material available online at https://doi.org/10.1155/2017/5310198) in absence and presence of outliers, respectively. Cleary we observe that, in absence of outliers, *β*-weight function produces larger weights and in presence of outliers, it produces smaller weights (almost close to zero) for unusual/outlying gene expressions. The outlier genes are indicated in red color (see Figure S1(b)). So we may conclude that our proposed method performs better in both situations, in absence and presence of outliers.

#### 3.2.2. Simulation Study 2

**Figure 2:**
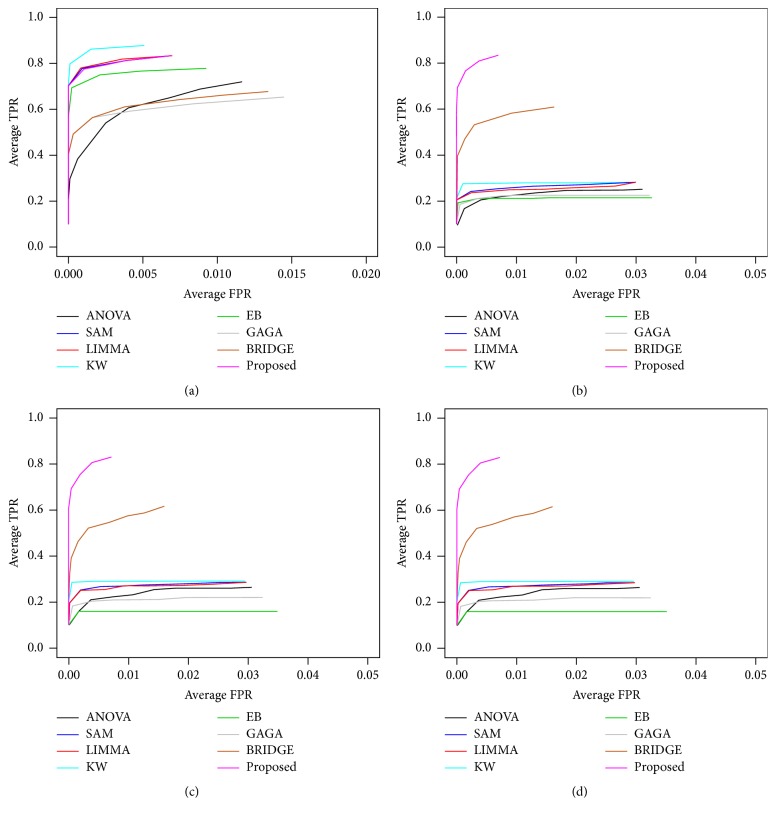
Performance evaluation using ROC curve produced by different methods for small-sample case (*n*_1_ = *n*_2_ = 3). (a) In absence of outliers. (b) One outlier with each of 10% genes. (c) One outlier with each of 20% genes. (d) One outlier with each of 50% genes.

**Figure 3:**
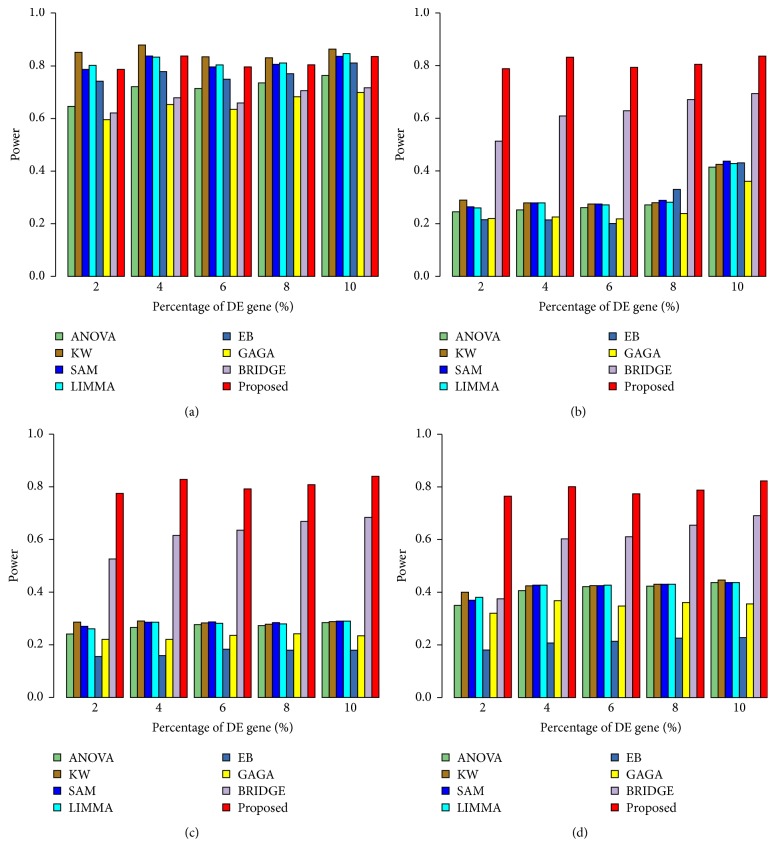
Performance evaluation using barplot of power estimated by different methods associated with varying proportions of DE gene. (a) In absence of outliers. (b) One outlier with each of 10% genes. (c) One outlier with each of 20% genes. (d) One outlier with each of 50% genes. Powers were estimated by eight methods (ANOVA, KW, SAM, LIMMA, EB, GAGA, BRIDGE, and proposed) based on top 200, 400, 600,800, and 1000 genes for small-sample case (*n*_1_ = *n*_2_ = 3).

**Figure 4:**
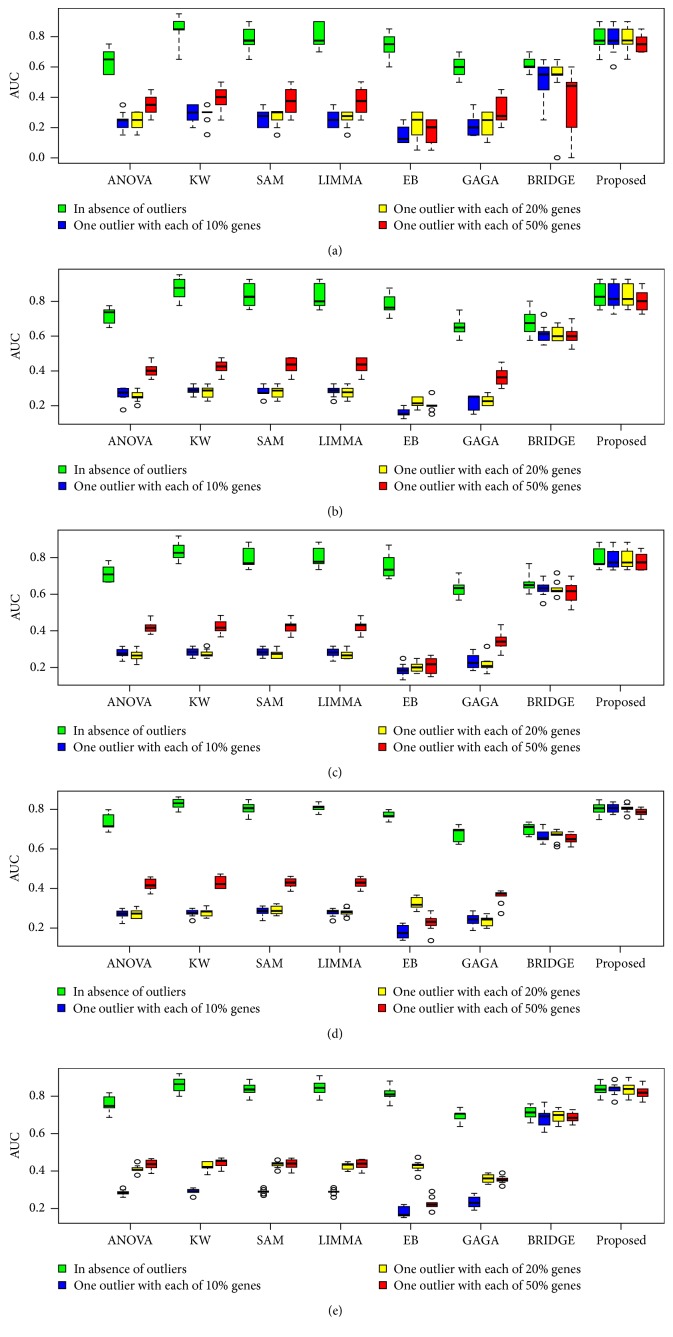
Performance evaluation using boxplot of AUC values estimated by different methods associated with varying proportions of DE gene. The panels (a), (b), (c), (d), and (e) represent the boxplot of AUC values estimated by eight methods (ANOVA, KW, SAM, LIMMA, EB, GAGA, BRIDGE, and proposed) for small-sample case (*n*_1_ = *n*_2_ = 3) at proportions of DE gene 0.02, 0.04, 0.06, 0.08, and 0.1, respectively, in absence and presence of one outlier in each of 10%, 20%, and 50% genes. 100 simulations were performed to obtain these results.

**Figure 5:**
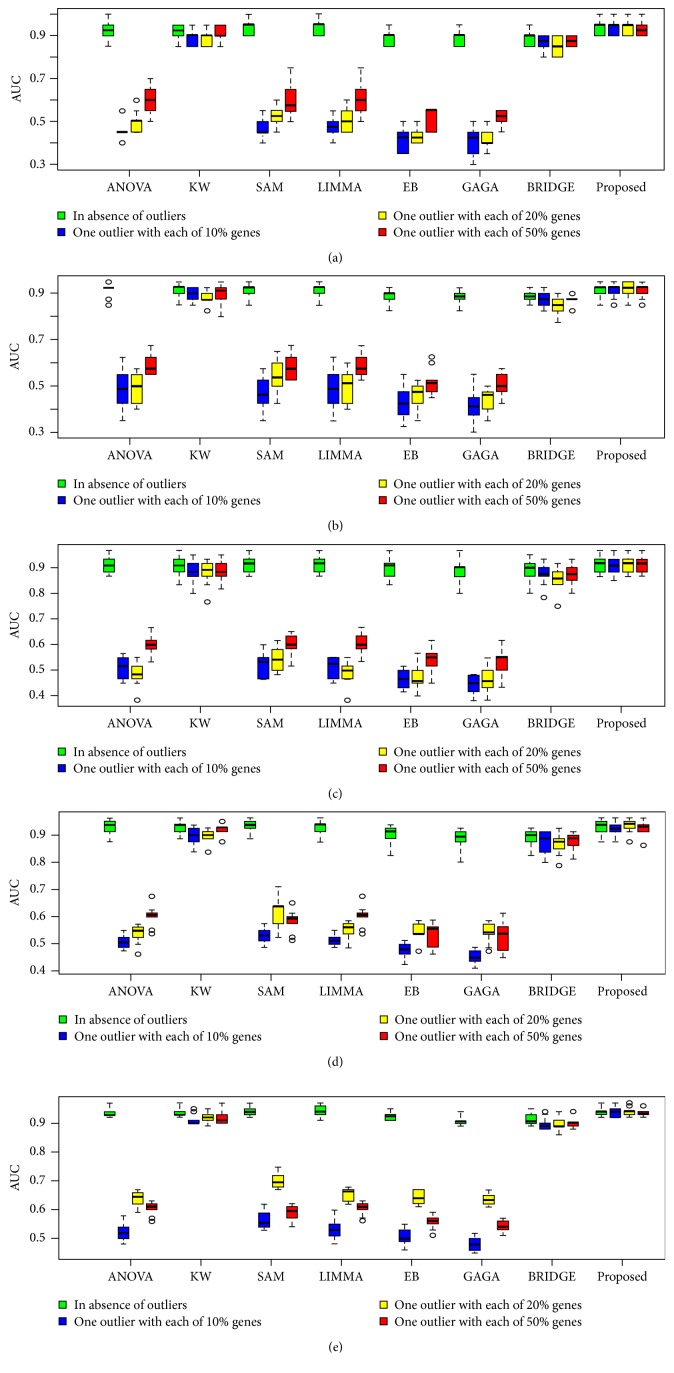
Performance evaluation using boxplot of AUC values estimated by different methods associated with varying proportions of DE gene. The panels (a), (b), (c), (d), and (e) represent the boxplot of AUC values estimated by eight methods (ANOVA, KW, SAM, LIMMA, EB, GAGA, BRIDGE, and proposed) for large-sample case (*n*_1_ = *n*_2_ = 25) at proportions of DE gene 0.02, 0.04, 0.06, 0.08, and 0.1, respectively, in absence and presence of one outlier in each of 10%, 20%, and 50% genes. 100 simulations were performed to obtain these results.

To investigate the performance of the proposed method in a comparison of the other seven popular methods as early mentioned for *k* = 2 conditions, we performed 100 simulations to generate 100 datasets for both small- (*n*_1_ = *n*_2_ = 3) and large- (*n*_1_ = *n*_2_ = 25) sample cases using (11). We set the arbitrary values (*μ*_*i*1_,*μ*_*i*2_)∈(3,5) and *σ*^2^ = 0.3 for datasets generated from each simulation. Each dataset for each case represented the gene expression profiles of *G* = 10,000 genes, with *n* = (*n*_1_ + *n*_2_) samples. The proportions of DE (pde) gene were set to 0.02, 0.04, 0.06, 0.08, and 0.1 for each of the 100 datasets. For these values, the theoretical numbers of DE genes are, respectively, 200, 400, 600, 800, and 1,000. To evaluate the performance of all the methods in presence of outliers, we generated 100 outlying datasets from each of the original datasets using (12). We consider one or two outliers in each of 10%, 20%, and 50% genes for each datasets. We computed average values of different performance measures such as TPR, TNR, FPR, FNR, FDR, AUC, and power based on 200, 400, 600, 800, and 1,000 estimated DE genes by eight methods (ANOVA, SAM, LIMMA, KW, EB, GAGA, BRIDGE, and proposed) for each of 100 datasets.  Figure 2 and Figure S2 show the ROC curve based on 400 estimated DE genes by each of the methods, in absence and presence of one or two outliers in each of 10%, 20%, and 50% genes for both small- and large-sample cases, respectively. We observe that all the eight methods performed almost similarly in absence of outliers for both small- and large-sample cases, except ANOVA for small-sample case (see Figure 2(a)). But in presence of outliers, the proposed method outperforms the other seven methods for small-sample case (see Figures 2(b)–2(d)). In this case BRIDGE also performs better. For large-sample case in presence of outliers, KW, BRIDGE, and proposed method perform well (see Figures S2(b)–S2(d)).  Figure 3 represents the barplot of power estimated by different methods associated with varying proportions of DE gene in both absence and presence of outliers for small-sample case. Figures 4 and 5 show the boxplots of AUC values based on 100 simulated datasets by each of the methods in absence and presence of outliers for small- and large-sample cases, respectively. The panels (a), (b), (c), (d), and (e) in Figures 4 and 5 show the boxplots of AUC values using the five values for parameter pde, 0.02, 0.04, 0.06, 0.08, and 0.1, respectively, for small- and large-sample cases. Similar results are found from these boxplots and barplots for all pde values like ROC curve.  Table 2 represents the average FDR estimated by eight methods based on 100 simulated datasets with pde 2%, 4%, 6%, 8%, and 10% in absence and presence of outliers for both small- and large-sample cases. In this table the results within the brackets (·), {·}, and [·] indicate estimated FDR by different methods in presence of one or two outliers in each of 10%, 20%, and 50% genes, respectively. From this table we also draw similar interpretations like ROC curve, boxplots, and barplots. So we may conclude that on an average the proposed method performed well compared to the other seven methods with *k* = 2 conditions both in absence and in presence of outliers.

#### 3.2.3. Simulation Study 3

To demonstrate the performance of the proposed method in a comparison of other popular methods for *k* > 2 conditions with multiple patterns, we generated 100 datasets for both small- (*n*_1_ = *n*_2_ = *n*_3_ = *n*_4_ = 3) and large- (*n*_1_ = *n*_2_ = *n*_3_ = *n*_4_ = 25) sample cases using (11). Each dataset was generated for (*μ*_*i*1_, *μ*_*i*2_, *μ*_*i*3_, *μ*_*i*4_) ∈ (3,5) and *σ*^2^ = 0.1. Each dataset contains the expression values for *G* = 10,000 genes with *n* = (*n*_1_ + *n*_2_ + *n*_3_ + *n*_4_) samples. The proportion of DE gene was fixed at 0.02 for each of the datasets. We generated the outlying gene expression datasets using (12) as before. We investigated the performance of the proposed method in a comparison of the other popular methods that are suitable for multiple-comparison tests (ANOVA, KW, SAM, and LIMMA). We first applied these methods to classify DE or EE genes and estimated different performance measures such as AUC, pAUC, MER, and FDR by these methods. Results obtained from these methods are presented in Table 3.

From this table we observe that, for small-sample case in absence of outliers, three methods (SAM, LIMMA, and proposed) exhibited better performance (AUC > 80%) than ANOVA and KW. KW performs worse in this case. But in presence of outliers, the proposed method outperforms other methods, producing more stable and consistent results (lower FDR and higher AUC, pAUC values). On the other hand, for large-sample case KW and proposed method performed well compared to the other methods (ANOVA, SAM, and LIMMA), in presence of outliers. Our proposed method exhibited slightly better performance than KW in this case, whereas in absence of outliers, they performed similarly. For both cases the Benjamini-Hochberg (BH) method was used to adjust the *p* values for each of the methods. Figure S3 represents the boxplots of MER values estimated by these five methods based on 200 DE genes in absence and presence of outliers for small-sample case. This figure also supports the results of Table 3. To demonstrate the pattern-detection performance of these methods, we again generated 100 datasets using (11). This time we consider the gene expression profiles for *G* = 1,000 genes with 300 DE genes for sample size of 3 in each condition. These 300 DE genes consist of four different patterns. These patterns are shown in Figure S4.  Table 4 represents the performance of different methods for detection of up- and downregulated genes in $\left(\begin{array}{c} 4\\ 2 \end{array}\right)=6$ pairs with *k* = 4 conditions for small-sample case. In this table the values within the bracket (*x*, *x*) represents the number of true up- and downregulated (UR, DR) DE genes in 6 pairs of conditions and the values within the brackets {*x*, *x*} and [*x*, *x*] represent the number of predicted (UR, DR) genes by five methods (ANOVA, KW, SAM, LIMMA, and proposed) and correctly detected number of (UR, DR) DE genes by these methods in absence and presence of one outlier in each of 10% genes. We observed that, in absence of outliers, three methods (SAM, LIMMA, and proposed) perform well for detecting of number of (UP, DR) genes in different pairs. KW performed badly in this case compared to ANOVA, whereas, in presence of outliers, the proposed method performed better than other methods (ANOVA, KW, SAM, and LIMMA). So, from this simulated study, we may conclude that the proposed method outperforms other methods in presence of outliers and in absence of outliers it keeps equal performance with other methods.

### 3.3. Real Microarray Data

To evaluate the performance of the proposed method in a comparison of the other seven methods as mentioned earlier, we used four microarray datasets. The first dataset is the Colon Cancer dataset [26] which consists of 22 control and 40 colon cancer samples. The second dataset is the Leukemia dataset [27]. The third dataset is the Platinum Spike dataset [28], which consists of 18 spike-in samples (9 controls versus 9 tests). The last dataset is the Breast Cancer dataset [29], which included 3226 genes measured on 22 breast cancer samples (7 sporadic, 7 BRCA-1, and 8 BRCA-2).

#### 3.3.1. Colon Cancer Microarray Dataset

**Figure 6:**
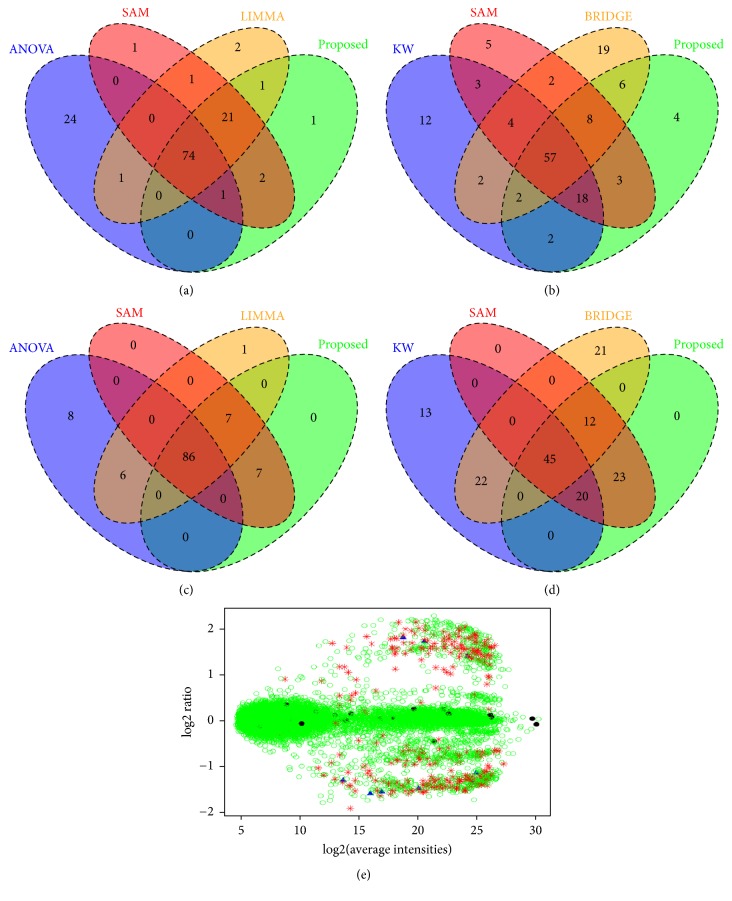
Comparison of the top selected genes by different methods for the Colon, Leukemia, and Platinum Spike datasets. Colon dataset, Venn diagram of top 100 genes estimated by (a) the ANOVA, SAM, LIMMA, and proposed method or by (b) the KW, SAM, BRIDGE, and proposed method. Leukemia dataset, Venn diagram of top 100 genes estimated by (c) the ANOVA, SAM, LIMMA, and proposed method or by (d) the KW, SAM, BRIDGE, and proposed method. Platinum Spike dataset, (e)* M*-*A* plot for randomly selected small-sample size (*n*_1_ = *n*_2_ = 3) from 9 controls and 9 tests samples in presence of one outlier in 20% of 1944 valid DE genes. The red asterisk, blue triangle, and black circle are used for 312, 9, and 40 genes detected by proposed method, LIMMA, and SAM that are common with valid set (see Figure S5(c)).

We used the colon cancer gene expression dataset. The dataset was downloaded from http://microarray.princeton.edu/oncology and was also used in the study [26]. The number of genes in this dataset is 2000.  Figure 6(a) represents the Venn diagram of top 100 genes estimated by ANOVA, SAM, LIMMA, and proposed method. From this Venn diagram, we clearly observe that our proposed method shares more genes with other methods. We further compared the performance of proposed method with two robust methods (KW and BRIDGE) and SAM (see Figure 6(b)). This comparison also revealed that proposed method shares more genes with SAM than KW or BRIDGE methods. There were 57 genes detected as common by these four methods. The proposed method also shared 18 genes with the SAM and KW methods, which were not detected by the BRIDGE method.

#### 3.3.2. Leukemia Microarray Dataset

This dataset was used in the study [27] and contains 7129 gene expressions for 72 leukemia samples in which 47 samples are acute lymphoblastic leukemia and 25 samples are acute myeloblastic leukemia. The results obtained from ANOVA, SAM, LIMMA, and proposed method based on top 100 estimated DE genes are presented in a Venn diagram in Figure 6(c). This figure shows that larger number of genes (86) is detected by these 4 methods. SAM and proposed method shared more genes (7) in this case. When comparing the proposed method with other robust methods (KW and BRIDGE) and SAM (see Figure 6(d)), we observed that 45 genes are common with these methods. The proposed method shares more genes with SAM than KW or BRIDGE methods. The proposed method also shared 20 genes with the SAM and KW methods, which were not detected by the BRIDGE method.

#### 3.3.3. Platinum Spike Microarray Dataset

We downloaded Affymetrix CEL format files from the GEO website with accession number GSE21344 and we applied robust multiarray average (RMA) to obtain signals for probes. The designated FC associated with these probes is downloaded from http://www.biomedcentral.com/content/supplementary/1471-2105-11-285-s5.txt. After RMA preprocessing, normalization, and dropout of the MC and MF values, we obtained 18707 probes. In this dataset the valid 1944 DE genes are known with different FC values. Then we applied the eight methods (ANOVA, KW, SAM, LIMMA, EB, GAGA, BRIDGE, and proposed) in this dataset to estimate different performance measures TPR, TNR, FPR, FNR, FDR, and AUC. We also investigate performance of all the methods in presence of outliers. We consider one outlier with 20% of valid DE genes using (12). The results are summarized in Table A1 in supporting file. From this table, we observed that all the eight methods perform almost similar, in absence of outliers, whereas in presence of outliers, BRIDGE and proposed method performed better than the other six methods (ANOVA, KW, SAM, LIMMA, EB, and GAGA). To evaluate the performance of these methods in the small number of samples, we selected the small subsets of samples from control and test group of patients. To select the subsamples we repeatedly took sample size 3 from 9 control and 9 test group patients and calculated the *p* value for each gene by these eight methods. This was repeated 100 times and the average of *p* values was recorded. The results are summarized in Table 5. We clearly observed that, in absence of outliers, three methods (SAM, LIMMA, and proposed) perform well compared to the other five methods (ANOVA, KW, EB, GAGA, and BRIDGE). But in presence of outliers, the proposed method outperformed other methods. Figures S5(a) and S5(b) shows Venn diagram of top 1944 genes detected by SAM, LIMMA, and proposed method and ANOVA, KW, and proposed method, respectively, with 1944 known valid DE genes' set, in absence of outliers. The Venn diagram in Figures S5(c) and S5(d) represents the top 1944 genes detected by the same methods, in presence of outliers. It is revealed from this Venn diagram that the proposed method performs better in both situations by sharing more genes with the valid 1944 DE genes.  Figure 6(e) shows the* M*-*A* plot for this dataset. The red asterisk, blue triangle, and black circle are used for 312, 9, and 40 genes detected by proposed method, LIMMA, and SAM, respectively, that are common with valid 1944 DE genes' set (see Figure S5(c)).

#### 3.3.4. BRCA Microarray Dataset

**Figure 7:**
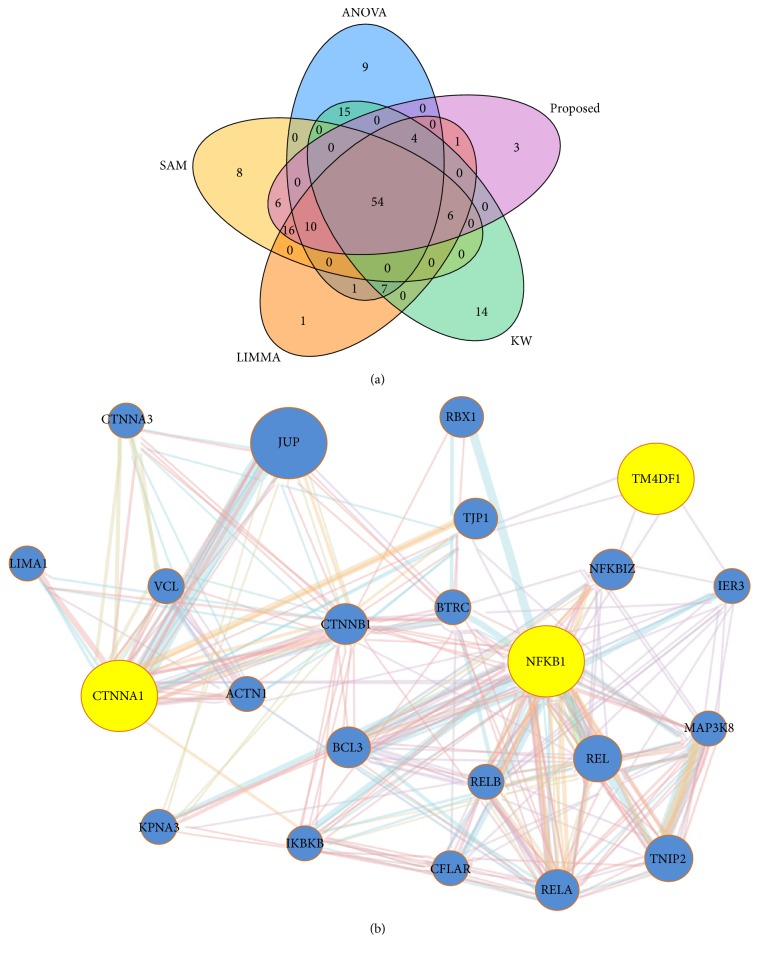
Comparison of the top 100 selected genes by five methods for the Breast Cancer dataset with three (*k* = 3) conditions. (a) Venn diagram of top 100 genes detected by ANOVA, KW, SAM, LIMMA, and proposed method. (b) Functional interactions of the three (3) genes detected by the proposed method were analyzed by GeneMANIA.

This data comes from the breast cancer cDNA microarray experiment [29]. This dataset consists of 3226 genes from 22 breast cancer samples, which are also divided into three classes (7 sporadic, 7 BRCA-1, and 8 BRCA-2) according to their mutational status.  Figure 7(a) represents the Venn diagram of top 100 genes identified by ANOVA, KW, SAM, LIMMA, and proposed method. From this Venn diagram we clearly observe that proposed method identified three genes that were not detected by the other methods (ANOVA, KW, SAM, and LIMMA). Then we explored the biological functions of these three genes (CTNNA1, NFKB1, and TM4SF1) using [30]. From this website we obtained GO (Gene Ontology), KEGG pathway, and disease association results. Using the GO database, we found that these genes are involved in biological processes and different molecular functions like negative regulation of programmed cell death, positive regulation of signal transduction, negative regulation of cell death, protein binding, protein complex, and molecular function (see S1 File (xls)). Figure S6 shows the Gene Ontology (GO) categories of these three (3) genes using directed acyclic graph (DAG). Using the KEGG database, we found that these genes are involved in cancer pathways with adjusted *p* value = 0.0002 (see Table 6 and S2 File (.xls)).  Table 7 represents the disease association results of these genes. In both tables the hypergeometric test is used to calculate the *p* values and adjusted by Benjamini-Hochberg method for multiple testing corrections.  Figure 7(b) represents the functional interactions (gene network) of these 3 genes were analyzed by GeneMANIA web server [31].

## 4. Conclusion

Differentially expressed (DE) genes identification to discover the disease biomarkers is one of the important tasks in microarray data analysis. Significance Analysis of Microarrays (SAM) is a popular statistical approach for identification of DE genes for both small- and large-sample cases. However, it is sensitive to outlying gene expressions and produces low power in presence of outliers. Therefore, in this paper, an attempt is made to robustify the SAM approach using the minimum *β*-divergence method. We used MLEs, in absence of outliers with *β* = 0 and in presence of outliers, the minimum *β*-divergence estimators are used to calculate the SAM statistic. We revealed from the simulated and real gene expression data analysis with *k* = 2 conditions that all the eight methods behave almost similar in absence of outliers, for both small- and large-sample cases. For large-sample case, in presence of outliers, three methods (KW, BRIDGE, and proposed) performed well compared to the other five methods (ANOVA, SAM, LIMMA, EB, and GAGA). However, the proposed method outperforms other seven methods for small-sample case, in presence of outliers. From the simulated dataset with *k* = 4 conditions with multiple patterns, we observed that five methods (ANOVA, KW, SAM, LIMMA, and proposed) perform well in absence of outliers, both small- and large-sample cases, except KW for small-sample case, whereas in presence of outliers for large-sample case two methods (KW and proposed) perform well. However, the proposed method outperforms the other four methods (ANOVA, KW, SAM, and LIMMA) for small-sample case, in presence of outliers. Similar results were found from real gene expression data analysis. Therefore, we may conclude that, on an average, the proposed method performs better than the other methods.

## Supplementary Material

Figure S1: Plot of smallest β-weight for simulation study 1. (a) In absence of outliers. (b) One outlier with each of 10% genes. Where the smallest β-weight represents the minimum value of 20 β-weights for 20 samples for each gene. The outlier genes are indicated in red color. The gray line indicates the maximum value of cutoff, λ = 0.13 for outlying genes.
Figure S2: Performance evaluation using ROC curve produced by different methods for large-sample case (*n*_1_ = *n*_2_ = 25). (a) In absence of outliers. (b) One outlier with each of 10% genes. (c) One outlier with each of 20% genes. (d) One outlier with each of 50% genes.
Figure S3: Performance evaluation using boxplot of MER values estimated by five methods for small-sample case (*n*_1_ = *n*_2_ = *n*_3_ = *n*_4_ = 3). Boxplot of MER values in absence and presence of one outlier with each of 10%, 20%, and 50% genes for small-sample case (*n*_1_ = *n*_2_ = *n*_3_ = *n*_4_ = 3). The MER values were calculated by five methods (ANOVA, KW, SAM, LIMMA, and proposed) based on top 200 genes.
Figure S4: Four different patterns of DE genes for small-sample case (*n*_1_ = *n*_2_ = *n*_3_ = *n*_4_ = 3) using simulation study 3.
Figure S5: Comparison of the top 1944 selected genes by five methods with 1944 valid DE gene set for Platinum Spike dataset for small-sample case (*n*_1_ = *n*_2_ = 3). In absence of outliers, Venn diagram of top 1944 genes detected by (a) the SAM, LIMMA, and proposed method or by (b) the ANOVA, KW, and proposed method with 1944 valid DE genes' set. In presence of one outlier in 20% of 1944 valid DE genes, Venn diagram of top 1944 genes detected by (c) the SAM, LIMMA, and proposed method or by (d) the ANOVA, KW, and proposed method with 1944 valid DE genes' set.
Figure S6: Gene Ontology (GO) categories of three (3) genes for BRCA dataset. The directed acyclic graph (DAG) shows the GO categories of three (3) genes, detected by the proposed method only for Breast Cancer (BRCA) dataset. In the DAG tree, each box in the tree lists holds the name of the GO category, the number of genes in the category, and adjusted *p* value. The box with red categories indicates that they are enriched with adj. *p* value <0.05.
Table A1: Performance evaluation of different methods based on Spike gene expression dataset.
S1 File: GO pathways for 3 breast cancer DE genes using BRCA dataset. Gene Ontology analysis information (XLS).
S2 File: KEGG pathways for 3 breast cancer DE genes using BRCA dataset. KEGG analysis information (XLS).

## Figures and Tables

**Table 1:** 

Predicted status	True status	
	DE	EE
DE	True positives (TP)	False positives (FP) (type I error)
EE	False negatives (FN) (type II error)	True negatives (TN)

**Table 2:** Performance evaluation of different methods using average values of FDR associated with varying proportions of DE gene.

Percentage of DE gene	ANOVA	KW	SAM	LIMMA	EB	GAGA	BRIDGE	Proposed
	Average FDR for small-sample case (*n*_1_ = *n*_2_ = 3)							

2%	0.355	0.205	0.215	0.210	0.260	0.405	0.380	0.215
	(0.755)	(0.710)	(0.735)	(0.740)	(0.785)	(0.780)	(0.485)	(0.210)
	{0.760}	{0.715}	{0.730}	{0.740}	{0.845}	{0.780}	{0.475}	{0.225}
	[0.650]	[0.600]	[0.630]	[0.620]	[0.820]	[0.680]	[0.625]	[0.235]

4%	0.280	0.152	0.165	0.168	0.222	0.347	0.322	0.165
	(0.748)	(0.720)	(0.720)	(0.720)	(0.785)	(0.775)	(0.390)	(0.168)
	{0.735}	{0.710}	{0.715}	{0.715}	{0.840}	{0.780}	{0.385}	{0.172}
	[0.595]	[0.575]	[0.572]	[0.572]	[0.792]	[0.632]	[0.398]	[0.200]

6%	0.287	0.197	0.205	0.198	0.252	0.367	0.342	0.200
	(0.738)	(0.725)	(0.725)	(0.728)	(0.798)	(0.782)	(0.372)	(0.207)
	{0.723}	{0.717}	{0.713}	{0.718}	{0.817}	{0.765}	{0.365}	{0.208}
	[0.578]	[0.575]	[0.575]	[0.573]	[0.787]	[0.653]	[0.390]	[0.227]

8%	0.266	0.181	0.196	0.191	0.231	0.318	0.296	0.196
	(0.728)	(0.720)	(0.710)	(0.719)	(0.669)	(0.761)	(0.329)	(0.195)
	{0.728}	{0.722}	{0.716}	{0.720}	{0.821}	{0.759}	{0.332}	{0.192}
	[0.578]	[0.570]	[0.570]	[0.570]	[0.774]	[0.640]	[0.346]	[0.210]

10%	0.239	0.152	0.165	0.156	0.190	0.303	0.285	0.165
	(0.585)	(0.574)	(0.563)	(0.571)	(0.569)	(0.639)	(0.305)	(0.163)
	{0.715}	{0.712}	{0.710}	{0.711}	{0.822}	{0.766}	{0.317}	{0.161}
	[0.564]	[0.553]	[0.563]	[0.563]	[0.773]	[0.646]	[0.310]	[0.178]

	Average FDR for large-sample case (*n*_1_ = *n*_2_ = 25)							

2%	0.075	0.080	0.060	0.060	0.110	0.115	0.110	0.060
	(0.500)	(0.105)	(0.470)	(0.495)	(0.570)	(0.580)	(0.145)	(0.065)
	{0.540}	{0.105}	{0.525}	{0.520}	{0.580}	{0.590}	{0.130}	{0.060}
	[0.405]	[0.095]	[0.410]	[0.400]	[0.485]	[0.480]	[0.125]	[0.070]

4%	0.082	0.087	0.085	0.085	0.115	0.117	0.115	0.085
	(0.510)	(0.115)	(0.458)	(0.500)	(0.540)	(0.560)	(0.148)	(0.082)
	{0.515}	{0.102}	{0.525}	{0.515}	{0.568}	{0.582}	{0.125}	{0.088}
	[0.408]	[0.102]	[0.420]	[0.410]	[0.480]	[0.498]	[0.125]	[0.088]

6%	0.090	0.092	0.088	0.086	0.101	0.111	0.11	0.088
	(0.515)	(0.118)	(0.453)	(0.510)	(0.530)	(0.533)	(0.145)	(0.088)
	{0.487}	{0.113}	{0.480}	{0.490}	{0.533}	{0.552}	{0.127}	{0.093}
	[0.400]	[0.113]	[0.403]	[0.398]	[0.460]	[0.467]	[0.125]	[0.087]

8%	0.070	0.070	0.06	0.072	0.096	0.115	0.109	0.069
	(0.460)	(0.104)	(0.384)	(0.450)	(0.459)	(0.459)	(0.134)	(0.069)
	{0.493}	{0.101}	{0.471}	{0.486}	{0.522}	{0.544}	{0.122}	{0.075}
	[0.398]	[0.081]	[0.416]	[0.398]	[0.465]	[0.471]	[0.120]	[0.075]

10%	0.062	0.063	0.057	0.057	0.075	0.091	0.087	0.059
	(0.361)	(0.079)	(0.299)	(0.347)	(0.355)	(0.363)	(0.104)	(0.060)
	{0.477}	{0.089}	{0.437}	{0.468}	{0.494}	{0.521}	{0.103}	{0.061}
	[0.395]	[0.080]	[0.409]	[0.396]	[0.443]	[0.458]	[0.098]	[0.063]

In this table the values within the brackets (·), {·}, and [·] represent the average values of FDR estimated by different methods (ANOVA, KW, SAM, LIMMA, EB, GAGA, BRIDGE, and proposed) in presence of one or two outliers in each of 10%, 20%, and 50% genes, respectively.

**Table 3:** Performance evaluation of different methods using AUC, pAUC, and FDR values for both small- and large-sample cases.

Performance measures	ANOVA	KW	SAM	LIMMA	Proposed
	For small-sample case (*n*_1_ = *n*_2_ = *n*_3_ = *n*_4_ = 3)				

AUC	0.764	0.196	0.832	0.834	0.832
	(0.279)	(0.102)	(0.192)	(0.279)	(0.839)
	{0.287}	{0.099}	{0.194}	{0.289}	(0.819)
	[0.084]	[0.009]	[0.177]	[0.097]	[0.839]

pAUC	0.152	0.038	0.166	0.166	0.166
	(0.055)	(0.019)	(0.038)	(0.055)	(0.167)
	{0.057}	{0.019}	{0.038}	{0.057}	{0.163}
	[0.016]	[0.019]	[0.035]	[0.019]	[0.167]

FDR	0.235	0.802	0.167	0.165	0.167
	(0.720)	(0.897)	(0.807)	(0.720)	(0.160)
	{0.712}	{0.900}	{0.805}	{0.710}	{0.180}
	[0.915]	[0.900]	[0.822]	[0.902]	[0.160]

	For large-sample case (*n*_1_ = *n*_2_ = *n*_3_ = *n*_4_ = 25)				

AUC	0.957	0.957	0.957	0.957	0.957
	(0.446)	(0.864)	(0.594)	(0.446)	(0.937)
	{0.432}	{0.874}	{0.614}	{0.432}	{0.947}
	[0.227]	[0.857]	[0.487]	[0.227]	[0.947]

pAUC	0.191	0.191	0.191	0.191	0.191
	(0.088)	(0.172)	(0.118)	(0.088)	(0.187)
	{0.086}	{0.174}	{0.122}	{0.086}	{0.189}
	[0.045]	[0.171]	[0.097]	[0.045]	[0.188]

FDR	0.042	0.042	0.045	0.042	0.042
	(0.552)	(0.135)	(0.405)	(0.552)	(0.062)
	{0.567}	{0.125}	{0.385}	{0.567}	{0.052}
	[0.772]	[0.145]	[0.512]	[0.772]	[0.057]

In this table the values within the brackets (·), {·}, and [·] represent the summary statistics estimated by different methods (ANOVA, KW, SAM, LIMMA, and proposed) in presence of one or two outliers in each of 10%, 20%, and 50% genes, respectively.

**Table 4:** Performance evaluation of different methods for detection of up- and downregulated genes in 6 pairs with *k* = 4 conditions for small-sample case (*n*_1_ = *n*_2_ = *n*_3_ = *n*_4_ = 3).

Methods	Pairwise comparison					
	1vs2	1vs3	1vs4	2vs3	2vs4	3vs4
	In absence of outliers					

*True (UR, DR)*	*(100, 30)*	*(100, 120)*	*(100, 50)*	*(30, 120)*	*(30, 50)*	*(120, 50)*
ANOVA	{75,20}	{72,85}	{75,45}	{24,83}	{26,43}	{89,40}
	[72,18]	[69,79]	[71,40]	[22,82]	[22,40]	[82,39]
SAM	{110,35}	{105,123}	{106,55}	{35,125}	{38,59}	{127,55}
	[95,30]	[93,111]	[93,49]	[29,114]	[30,49]	[114,48]
LIMMA	{86,28}	{98,112}	{90,48}	{28,113}	{30,47}	{114,49}
	[85,27]	[92,107]	[87,47]	[26,108]	[27,45]	[108,45]
KW	{25,12}	{23,33}	{30,25}	{12,35}	{12,17}	{36,19}
	[18,6]	[19,28]	[22,16]	[5,29]	[5,15]	[32,13]
Proposed	{106, 34}	{104, 125}	{105, 56}	{36, 127}	{37, 56}	{128, 56}
	[93, 29]	[93, 116]	[100, 49]	[30, 116]	[30, 49]	[117, 50]

	In presence of one outlier with each of 10% genes					

*True (UR, DR)*	*(100, 30)*	*(100, 120)*	*(100, 50)*	*(30, 120)*	*(30, 50)*	*(120, 50)*
ANOVA	{28,10}	{28,40}	{27,11}	{12,37}	{11,18}	{37,14}
	[27, 8]	[25,38]	[25,11]	[9,36]	[9,17]	[34,12]
SAM	{75,35}	{78,90}	{74,50}	{28,82}	{31,40}	{84,38}
	[62,19]	[65,75]	[59,31]	[17,72]	[17,26]	[74,29]
LIMMA	{43,23}	{42,62}	{44,26}	{20,56}	{20,26}	{61,30}
	[37,13]	[32,52]	[35,19]	[12,50]	[13,20]	[49,19]
KW	{23,9}	{13,19}	{22,10}	{6,21}	{6,13}	{22,13}
	[21,4]	[10,17]	[16,5]	[4,18]	[3,9]	[17, 9]
Proposed	{109, 33}	{111, 119}	{104, 56}	{35, 116}	{41, 59}	{122, 61}
	[86, 27]	[87, 113]	[83, 47]	[23, 105]	[26, 45]	[113, 49]

In this table the values within the bracket (*x*, *x*) represent the number of true up- and downregulated (UR, DR) DE genes in 6 pairs and the values within the brackets {*x*, *x*} and [*x*, *x*] represent the number of predicted (UR, DR) genes by five methods (ANOVA, KW, SAM, LIMMA, and proposed) and correctly detected number of (UR, DR) DE genes by these methods in both absence and presence of one outlier with each of 10% genes.

**Table 5:** Performance evaluation of different methods based on Spike gene expression dataset for randomly chosen subsamples of size 3 from each condition.

Methods	TPR	TNR	FPR	FNR	FDR	AUC
	In absence of outliers					

ANOVA	0.787	0.975	0.024	0.213	0.213	0.784
KW	0.788	0.975	0.024	0.211	0.219	0.781
SAM	0.821	0.979	0.020	0.178	0.175	0.820
LIMMA	0.816	0.978	0.021	0.183	0.186	0.814
EB	0.644	0.958	0.041	0.356	0.356	0.642
GAGA	0.598	0.953	0.046	0.401	0.407	0.595
BRIDGE	0.644	0.958	0.041	0.355	0.355	0.642
Proposed	0.821	0.978	0.021	0.185	0.175	0.820

	In presence of one outlier in 20% of 1944 DE genes					

ANOVA	0.630	0.957	0.043	0.370	0.370	0.622
KW	0.620	0.955	0.044	0.380	0.380	0.611
SAM	0.730	0.968	0.031	0.270	0.270	0.726
LIMMA	0.720	0.967	0.032	0.284	0.284	0.712
EB	0.114	0.897	0.102	0.885	0.885	0.111
GAGA	0.608	0.954	0.045	0.392	0.392	0.605
BRIDGE	0.668	0.961	0.038	0.331	0.331	0.665
Proposed	0.806	0.977	0.022	0.193	0.193	0.805

In this table the summary statistics (TPR, TNR, FPR, FNR, FDR, and AUC) were estimated by different methods (ANOVA, KW, SAM, LIMMA, EB, GAGA, BRIDGE, and proposed) based on valid 1944 DE genes in both absence and presence of one outlier in 20% of valid 1944 DE genes.

**Table 6:** KEGG pathways for the three (3) DE genes identified by the proposed method only.

KEGG ID	KEGG pathway description	Number of genes	*p* value	Adjusted *p* value
hsa05200	Pathways in cancer	2	0.0002	0.0002

The hypergeometric test is used to calculate the *p* values and adjusted by Benjamini-Hochberg method for multiple testing corrections.

**Table 7:** Disease association results of three (3) genes identified by proposed method only.

Disease	Name of genes	Raw *p* values	Adj. *p* values
Neoplasm invasiveness	CTNNA1, NFKB1, TM4SF1	3.04*e* − 07	9.81*e* − 07
Neoplastic processes	CTNNA1, NFKB1	0.0003	0.0005
Adhesion	CTNNA1, NFKB1	0.0007	0.0007

The hypergeometric test is used to calculate the *p* values and adjusted by Benjamini-Hochberg method for multiple testing corrections.
