# Supplementary material for: Robust Significance Analysis of Microarrays by Minimum β-Divergence Method
Source: Biomed Res Int. 2017 Jul 27;2017:5310198. doi: 10.1155/2017/5310198 (PMC5551475; doi:10.1155/2017/5310198)
Supplement: Supplementary file 1 — Figure S1: Plot of smallest β-weight for simulation study 1. (a) In absence of outliers. (b) One outlier with each of 10% genes. Where the smallest β-weight represents the minimum value of 20 β-weights for 20 samples for each gene. The outlier genes are indicated in red color. The gray line indicates the maximum value of cutoff, λ = 0.13 for outlying genes. Figure S2: Performance evaluation using ROC curve produced by different methods for large-sample case (n1 = n2 = 25). (a) In absence of outliers. (b) One outlier with each of 10% genes. (c) One outlier with each of 20% genes. (d) One outlier with each of 50% genes. Figure S3: Performance evaluation using boxplot of MER values estimated by five methods for small-sample case (n1 = n2 = n3 = n4 = 3). Boxplot of MER values in absence and presence of one outlier with each of 10%, 20%, and 50% genes for small-sample case (n1 = n2 = n3 = n4 = 3). The MER values were calculated by five methods (ANOVA, KW, SAM, LIMMA, and proposed) based on top 200 genes. Figure S4: Four different patterns of DE genes for small-sample case (n1 = n2 = n3 = n4 = 3) using simulation study 3. Figure S5: Comparison of the top 1944 selected genes by five methods with 1944 valid DE gene set for Platinum Spike dataset for small-sample case (n1 = n2 = 3). In absence of outliers, Venn diagram of top 1944 genes detected by (a) the SAM, LIMMA, and proposed method or by (b) the ANOVA, KW, and proposed method with 1944 valid DE genes' set. In presence of one outlier in 20% of 1944 valid DE genes, Venn diagram of top 1944 genes detected by (c) the SAM, LIMMA, and proposed method or by (d) the ANOVA, KW, and proposed method with 1944 valid DE genes' set. Figure S6: Gene Ontology (GO) categories of three (3) genes for BRCA dataset. The directed acyclic graph (DAG) shows the GO categories of three (3) genes, detected by the proposed method only for Breast Cancer (BRCA) dataset. In the DAG tree, each box in the tree lists holds the name of the GO category, [file 5310198.f1.docx]

**Figure S1. Plot of smallest β-weight for simulation study 1.** (a) In absence of outliers. (b) In presence of one outlier in each of 10% genes. Where the smallest β-weight represents the minimum value of 20 β-weights for 20 samples for each gene. The outlier genes are indicated in red color. The gray line indicate the maximum value of cutoff, λ=0.13 for outlying genes.


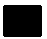
 Without outlier genes


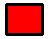
 With outlier genes


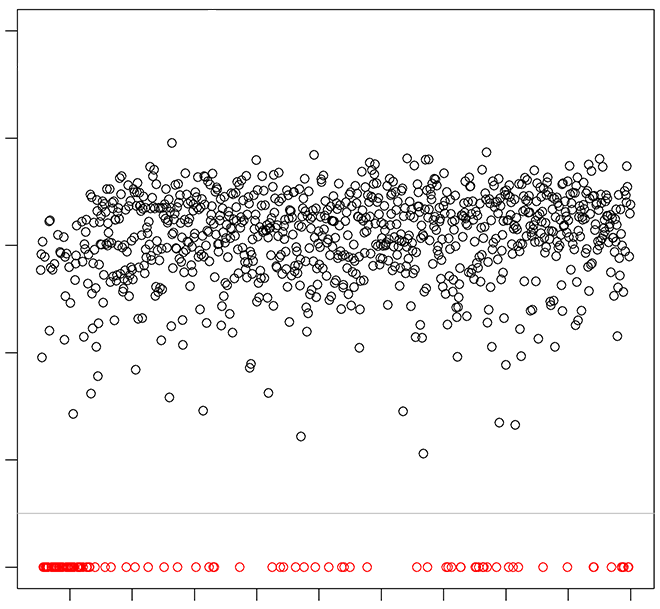


(b) Smallest β-weight with one outlier in each of 10% gene

1.0

0.8

0.6

0.4

0.2

0.0

Smallest β-weight

100

200

300

400

500

600

700

800

900

1000


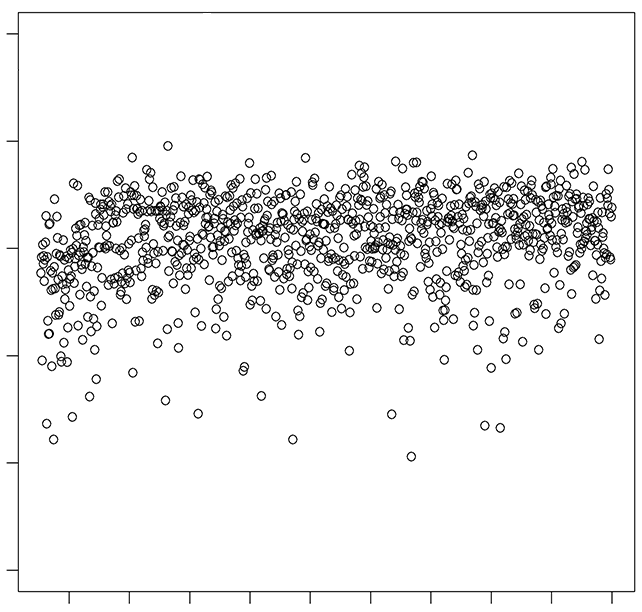


(a) Smallest β-weight without outlier

Smallest β-weight

1.0

0.8

0.6

0.4

0.2

0.0

100

200

300

400

500

600

700

800

900

1000
