# Supplementary material for: Robust Significance Analysis of Microarrays by Minimum β-Divergence Method
Source: Biomed Res Int. 2017 Jul 27;2017:5310198. doi: 10.1155/2017/5310198 (PMC5551475; doi:10.1155/2017/5310198)
Supplement: Supplementary file 2 [file 5310198.f2.docx]

Average FPR

(b) In presence of one outlier with each of 10% genes

Average TPR

1.0

0.8

0.6

0.4

0.2

0.0

0.00

0.01

0.02

0.03

0.04

0.05


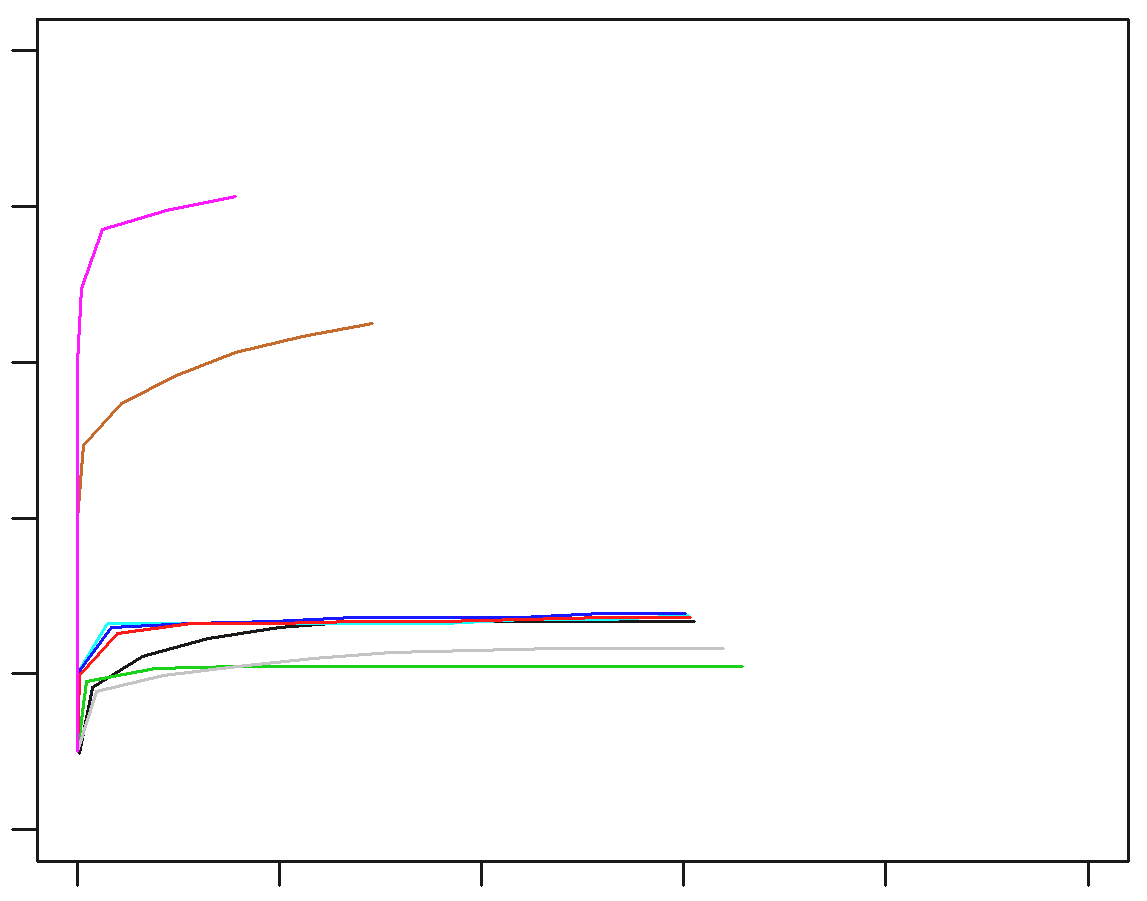


Average FPR

Average TPR

0.0

0.2

0.4

0.6

0.8

1.0

0.0001

0.005

0.010

0.015

0.020

(a) In Absence of Outliers


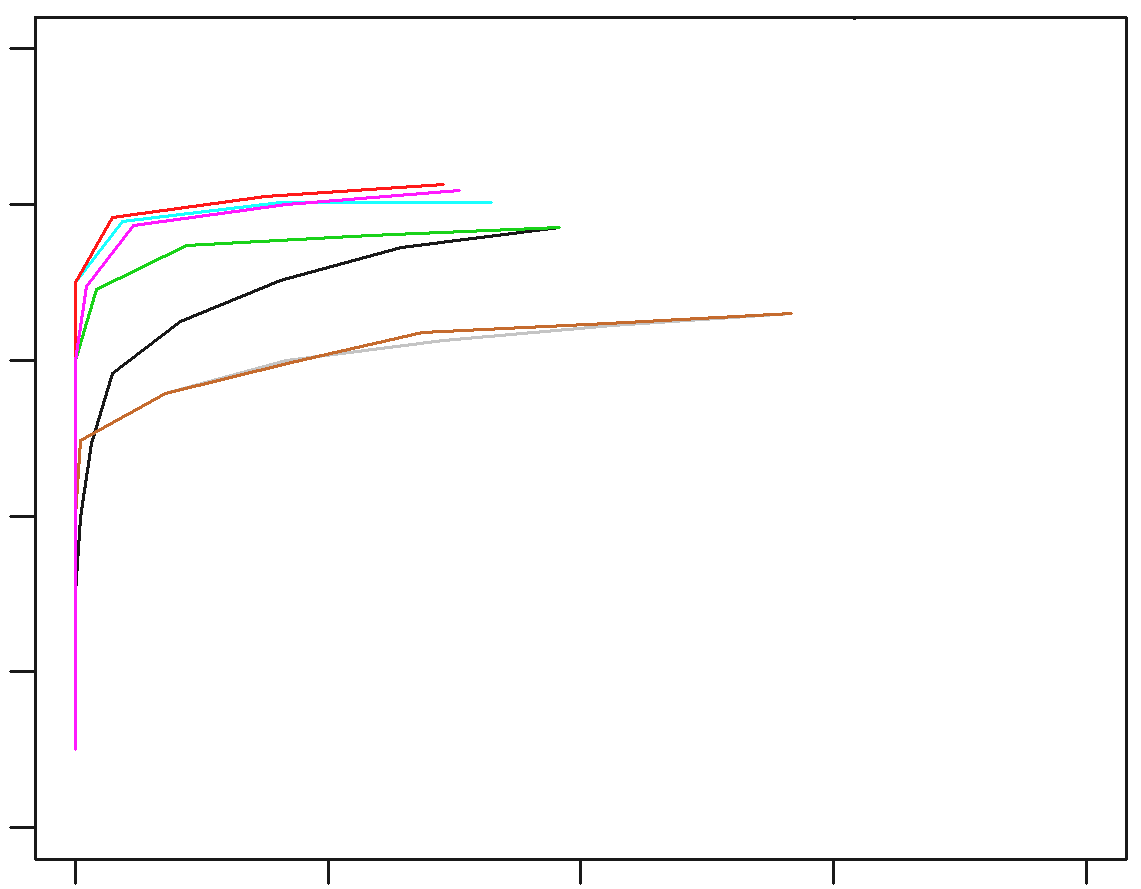


SAM

ANOVA

KW

LIMMA

KW

EB

BRIDGE

GAGA

Proposed

(d) In presence of one outlier with each of 50% genes

Average TPR

0.00

0.01

0.02

0.03

0.04

0.05

1.0

0.8

0.6

0.4

0.2

0.0


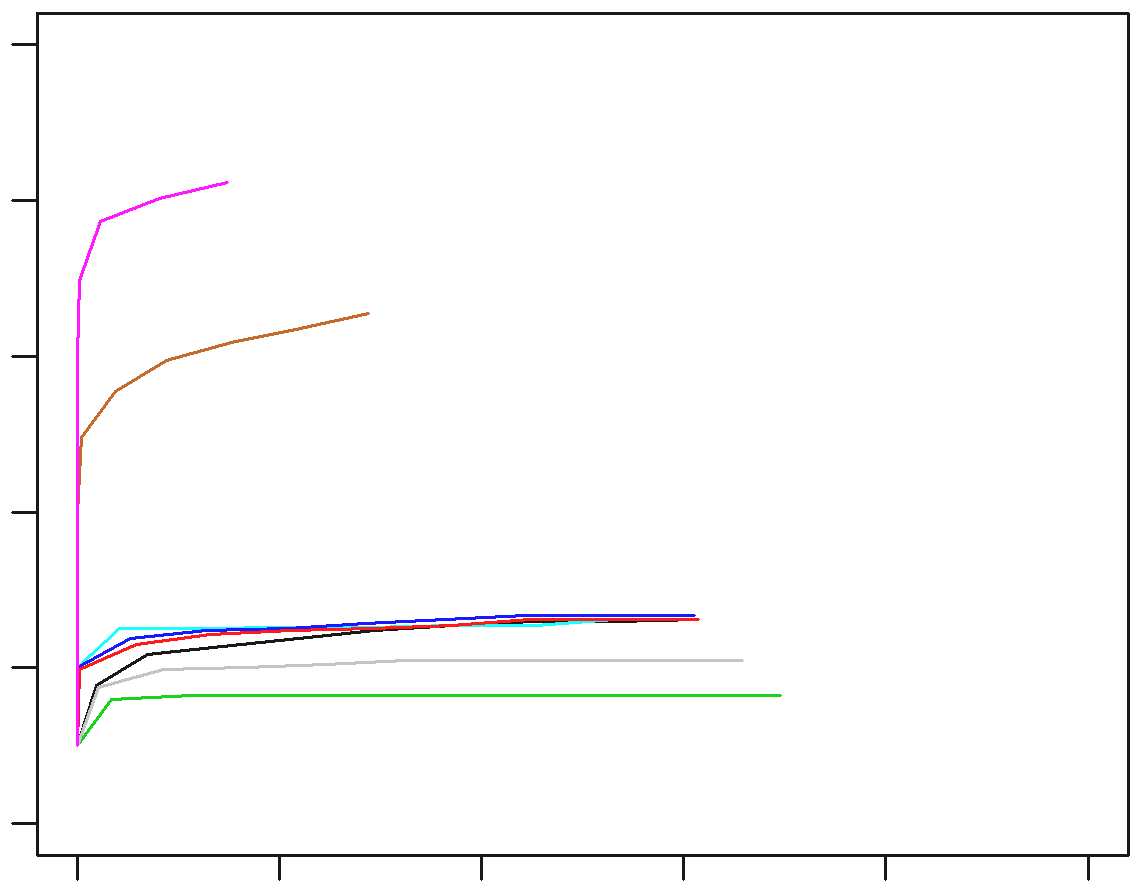


(c) In presence of one outlier with each of 10% genes

Average TPR

Average FPR

0.00

0.01

0.02

0.03

0.04

0.05

0.0

0.2

0.4

0.6

0.8

1.0


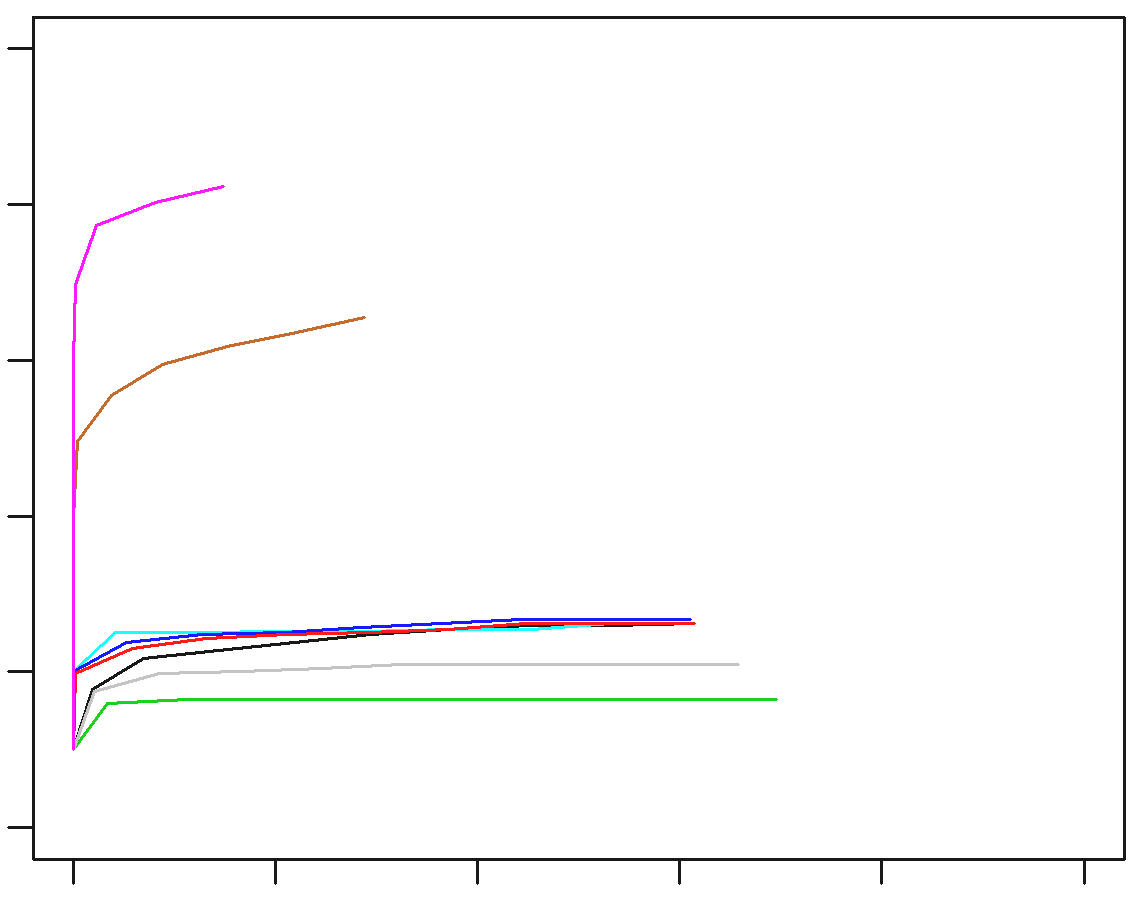


Average FPR

**Figure S2. Performance evaluation using ROC curve produced by different methods with large-sample case (*n*_1_ = *n*_2_ = 25) .** (a) In absence of outliers. (b) In presence of one outlier in each of 10% genes (c) In presence of one outlier in each of 20% genes. (d) In presence of one outlier in each of 50% genes.
