# Supplementary material for: Robust Significance Analysis of Microarrays by Minimum β-Divergence Method
Source: Biomed Res Int. 2017 Jul 27;2017:5310198. doi: 10.1155/2017/5310198 (PMC5551475; doi:10.1155/2017/5310198)
Supplement: Supplementary file 3 [file 5310198.f3.docx]

0.00

0.01

0.02

0.03

0.04


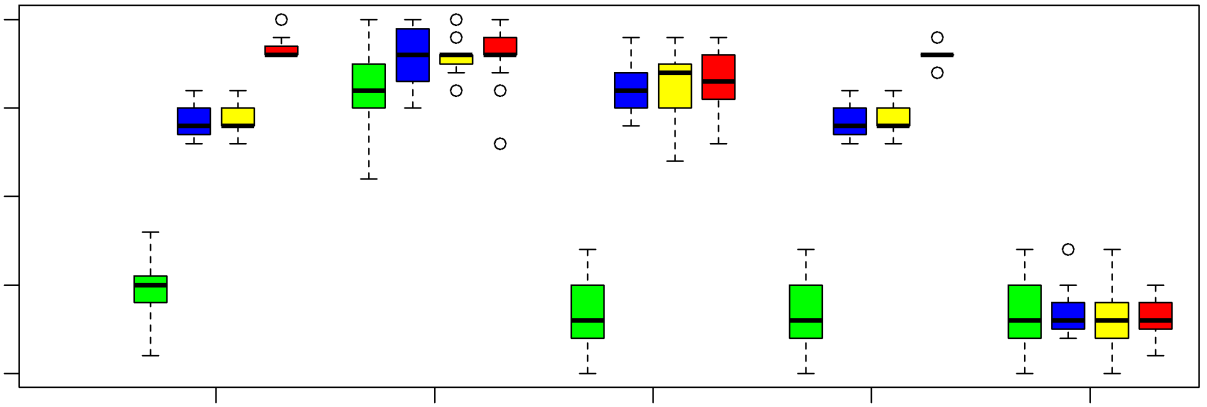


MER

ANOVA

KW

SAM

LIMMA

Proposed

Without Outliers

Outliers in 10% genes

Outliers in 20% genes

Outliers in 50% genes

**Figure S3. Performance evaluation using boxplot of MER values estimated by five methods for small-sample case (*n*_1_=*n*_2_= *n*_3_=*n*_4_=3)**. Boxplot of MER values in absence and presence of one outlier in each of 10%, 20% and 50% genes for small-sample case (*n*_1_=*n*_2_= *n*_3_=*n*_4_=3). The MER values were calculated by five methods (ANOVA, KW, SAM, LIMMA and Proposed) based on top 200 genes.
