# Supplementary material for: Robust Significance Analysis of Microarrays by Minimum β-Divergence Method
Source: Biomed Res Int. 2017 Jul 27;2017:5310198. doi: 10.1155/2017/5310198 (PMC5551475; doi:10.1155/2017/5310198)
Supplement: Supplementary file 4 [file 5310198.f4.docx]

Expressions


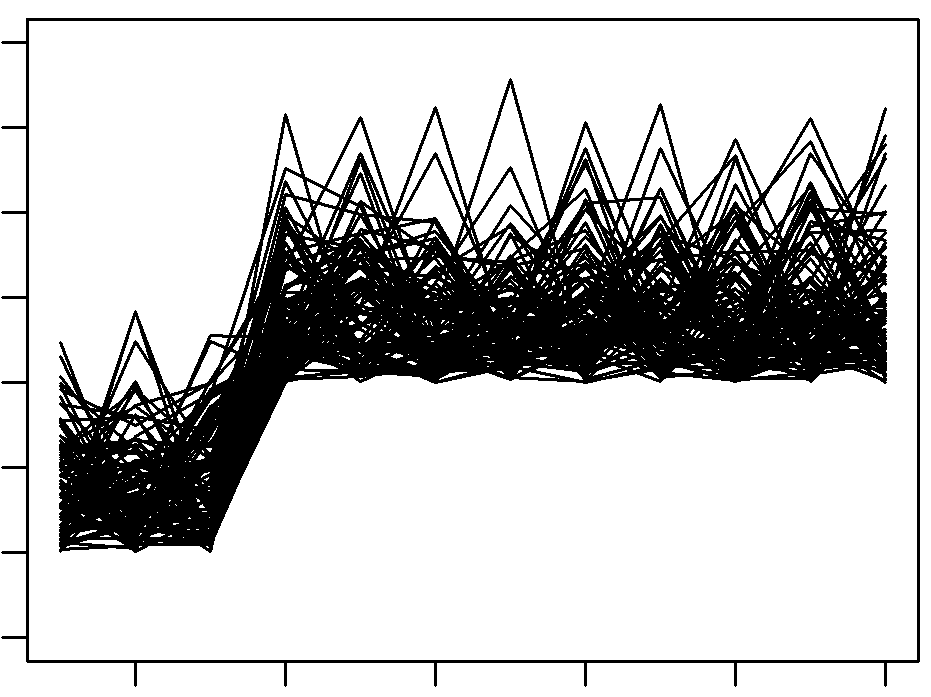


No. of Samples

(a)

-2

-1

0

1

2

3

-3

4

2

4

6

8

10

12

(b)

Expressions


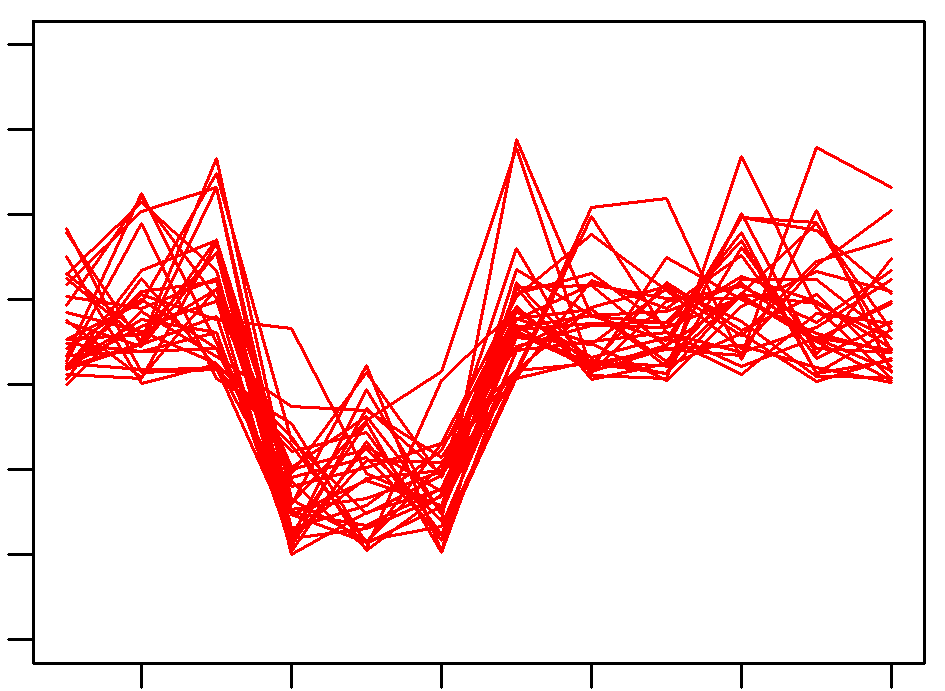


No. of Samples

4

3

2

1

0

-1

-2

-3

2

4

6

8

10

12

(d)


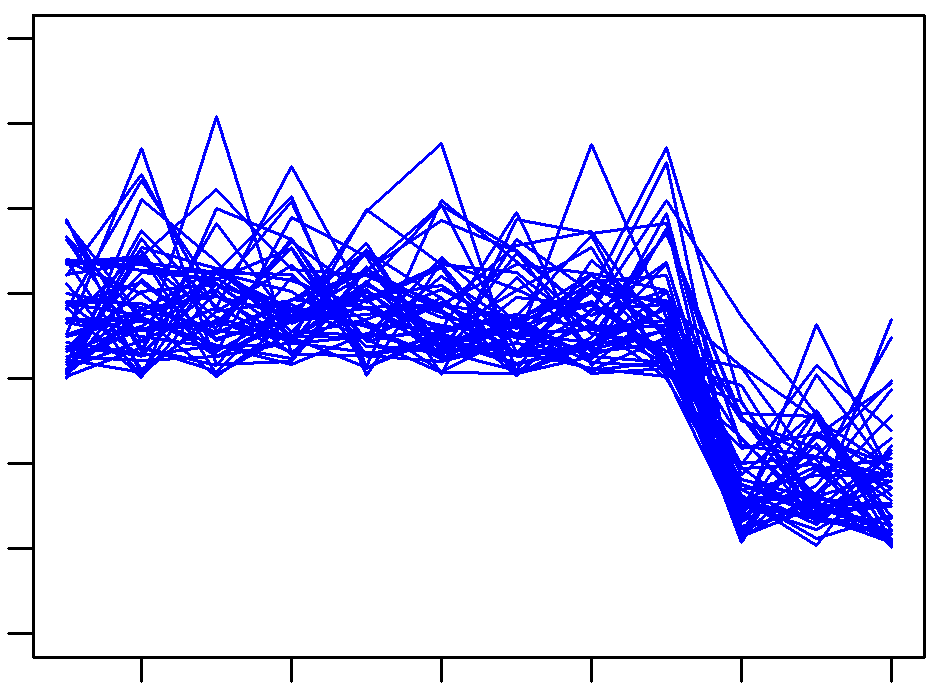


No. of Samples

Expressions

4

1

0

-1

-2

-3

2

3

2

4

6

8

10

12

2

4

6

8

10

12

(c)

Expressions

-3

-2

-1

0

1

3

2

4


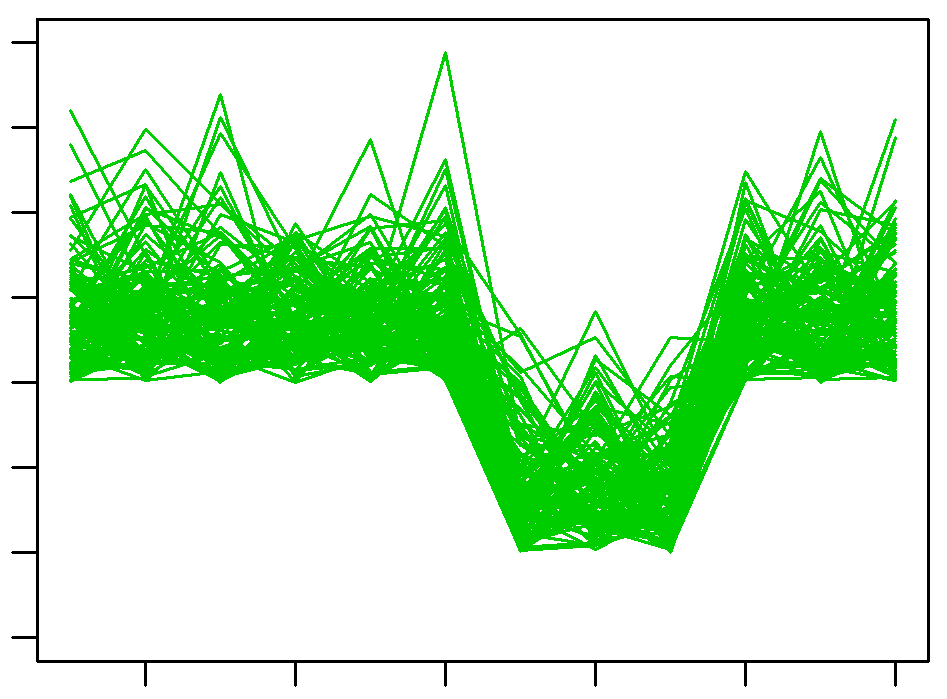


No. of Samples

**Figure S4. Four different patterns of DE genes for small-sample case (*n*_1_=*n*_2_= *n*_3_=*n*_4_=3)**.
