# Supplementary material for: Robust Significance Analysis of Microarrays by Minimum β-Divergence Method
Source: Biomed Res Int. 2017 Jul 27;2017:5310198. doi: 10.1155/2017/5310198 (PMC5551475; doi:10.1155/2017/5310198)
Supplement: Supplementary file 5 [file 5310198.f5.docx]

**Figure S5. Comparison of the top 1944 selected genes by five methods with 1944 valid DE gene set for Platinum Spike dataset for small-sample case (*n*_1_=*n*_2_=3)**. In absence of outliers, Venn diagram of top 1944 genes detected by (a) the SAM, LIMMA and Proposed method or by (b) the ANOVA, KW and Proposed method with 1944 valid DE gene set. In presence of one outlier in 20% of 1944 valid DE genes , Venn diagram of top 1944 genes detected by (c) the SAM, LIMMA and Proposed method or by (d) the ANOVA, KW and Proposed method with 1944 valid DE gene set.

Valid Set

SAM

LIMMA

Proposed

248

1097

82

302

2

9

32

294

126

51

40

312

124

4

21


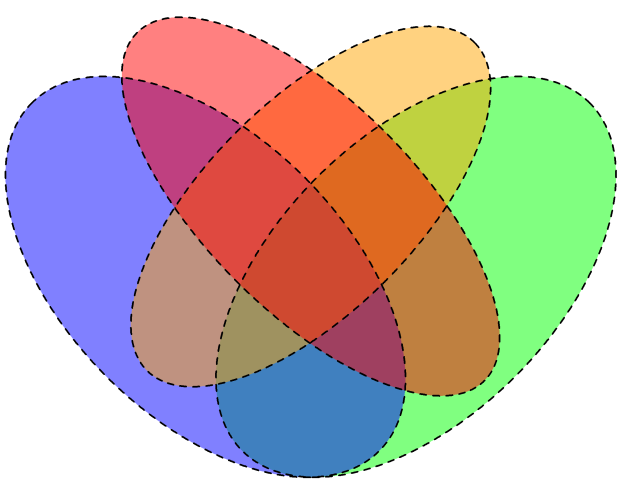


(c)

Valid Set

ANOVA

KW

Proposed

390

1117

47

214

69

21

57

211

208

58

8

74

230

49

144


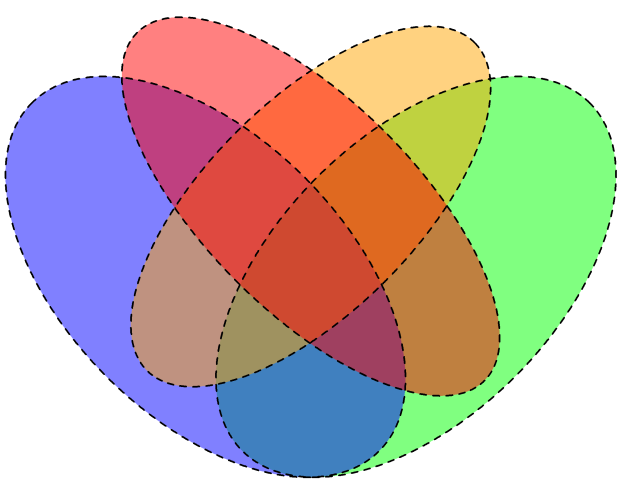


(b)

435

1417

2

361

28

4

24

66

42

27

13

29

20

2

71

Valid Set

SAM

LIMMA

Proposed


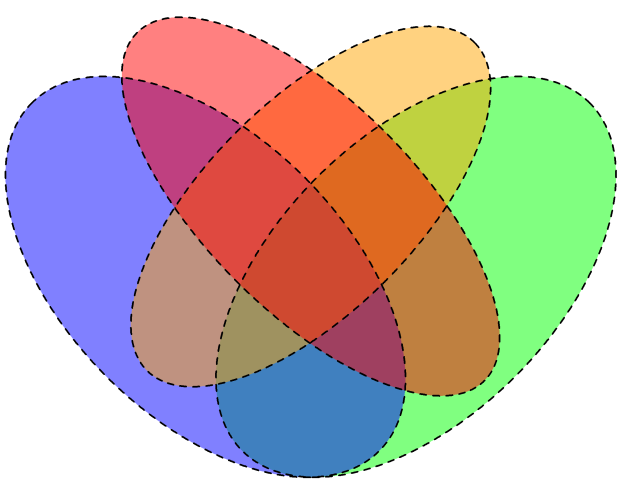


(a)

277

982

2

259

6

9

35

442

209

164

8

606

25

29

35

Valid Set

ANOVA

KW

Proposed


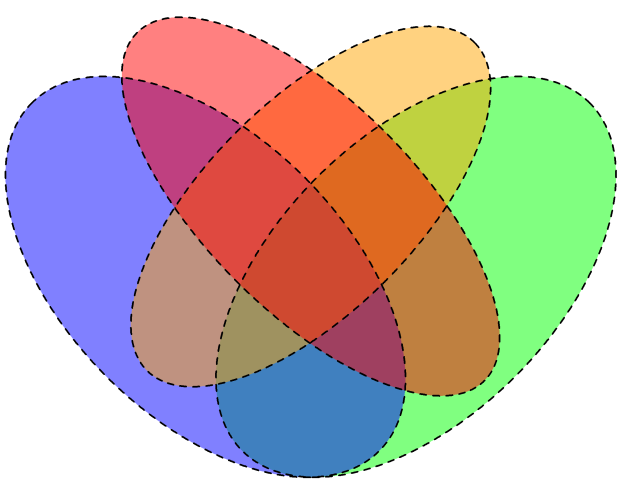


(d)
