# Supplementary material for: Robust Significance Analysis of Microarrays by Minimum β-Divergence Method
Source: Biomed Res Int. 2017 Jul 27;2017:5310198. doi: 10.1155/2017/5310198 (PMC5551475; doi:10.1155/2017/5310198)
Supplement: Supplementary file 6 [file 5310198.f6.docx]

biological_process

biological

regulation

single-organism

process

signaling

single

organism

signaling

regulation

of

signaling

negative

regulation

of

signaling

2 genes

adjP=4.70e-02

response

to

chemical

stimulus

3 genes

adjP=4.70e-02

response

to

drug

3 genes

adjP=4.70e-02

negative

regulation

of

response

to

stimulus

2 genes

adjP=4.70e-02

regulation

of

response

to

stimulus

regulation

of

signal

transduction

negative

regulation

of

signal

transduction

2 genes

adjP=4.70e-02

response

to

stimulus

regulation

of

biological

process

negative

regulation

of

biological

process

cellular

response

to

stimulus

signal

transduction

cell

communication

regulation

of

cellular

process

regulation

of

developmental

process

cell

cycle

cellular

developmental

process

cell

differentiation

mitotic

cell

cycle

2 genes

adjP=4.70e-02

regulation

of

cell

cycle

2 genes

adjP=4.70e-02

negative

regulation

of

developmental

process

2 genes

adjP=4.70e-02

negative

regulation

of

cellular

process

regulation

of

cell

communication

developmental

process

cellular

process

regulation

of

cell

differentiation

regulation

of

mitotic

cell

cycle

2 genes

adjP=4.70e-02

negative

regulation

of

cell

cycle

2 genes

adjP=4.70e-02

negative

regulation

of

cell

communication

2 genes

adjP=4.70e-02

negative

regulation

of

cell

cycle

differentiation

2 genes

adjP=4.70e-02


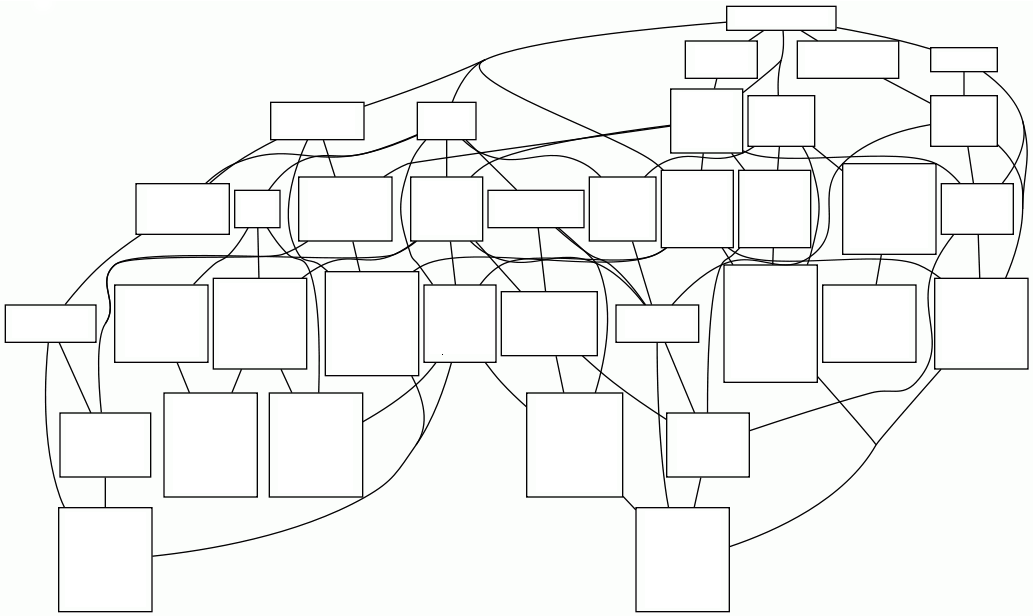


**Figure S6. Gene ontology (GO) categories of three (3) genes for BRCA dataset**. The directed acyclic graph (DAG) shows the GO categories of three (3) genes, detected by the Proposed method only for breast cancer (BRCA) dataset. In the DAG tree; each box in the tree lists the name of the GO category, the number of genes in the category, and adjusted *p*-value. The box with red categories indicates that they are enriched with adj. *p*-value<0.05.
