# Supplementary material for: Robust Significance Analysis of Microarrays by Minimum β-Divergence Method
Source: Biomed Res Int. 2017 Jul 27;2017:5310198. doi: 10.1155/2017/5310198 (PMC5551475; doi:10.1155/2017/5310198)
Supplement: Supplementary file 7 [file 5310198.f7.pdf]

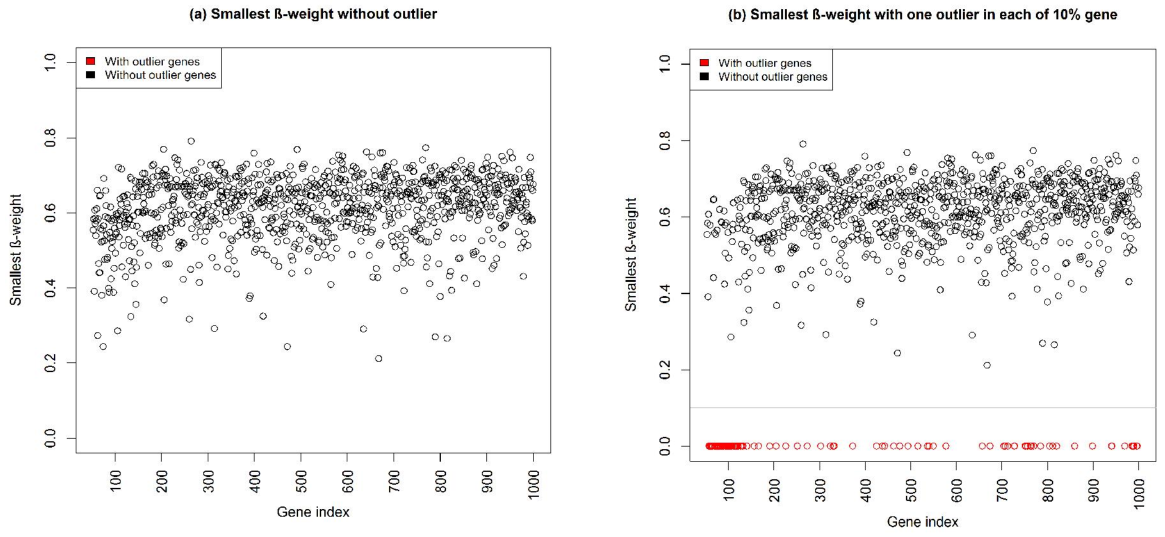

Figure S1. Plot of smallest β-weight for simulation study 1. (a) In absence of outliers. (b) In presence of one outlier in each of 10% genes. Where the smallest β-weight represents the minimum value of 20 β-weights for 20 samples for each gene. The outlier genes are indicated in red color. The gray line indicate the maximum value of cutoff, λ=0.13 for outlying genes.

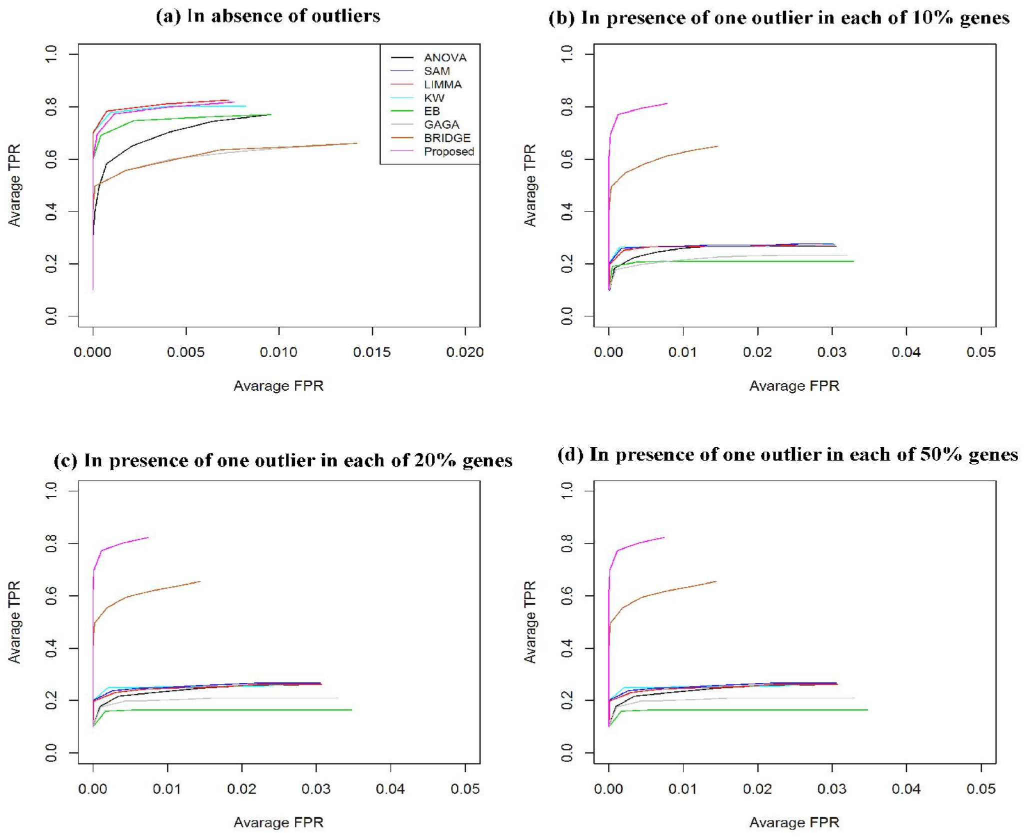

Figure S2. Performance evaluation using ROC curve produced by di□erent methods with large-sample case (n_1 = n_2 = 25) . (a) In absence of outliers. (b) In presence of one outlier in each of 10% genes (c) In presence of one outlier in each of 20% genes. (d) In presence of one outlier in each of 50% genes.

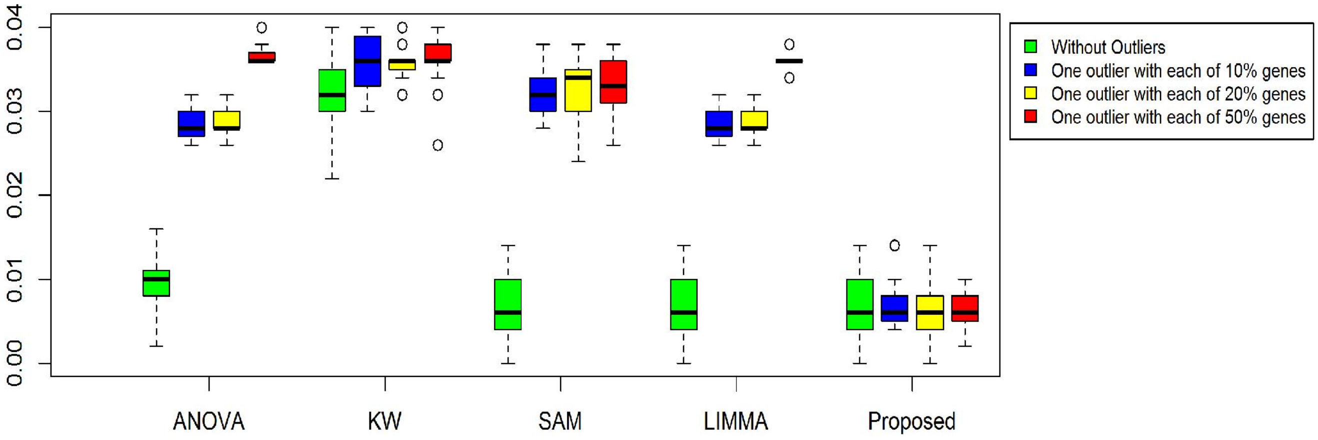

Figure S3. Performance evaluation using boxplot of MER values estimated by five methods for small-sample case (n_1=n_2= n_3=n_4=3). Boxplot of MER values in absence and presence of one outlier in each of 10%, 20% and 50% genes for small-sample case (n_1=n_2= n_3=n_4=3). The MER values were calculated by five methods (ANOVA, KW, SAM, LIMMA and Proposed) based on top 200 genes.

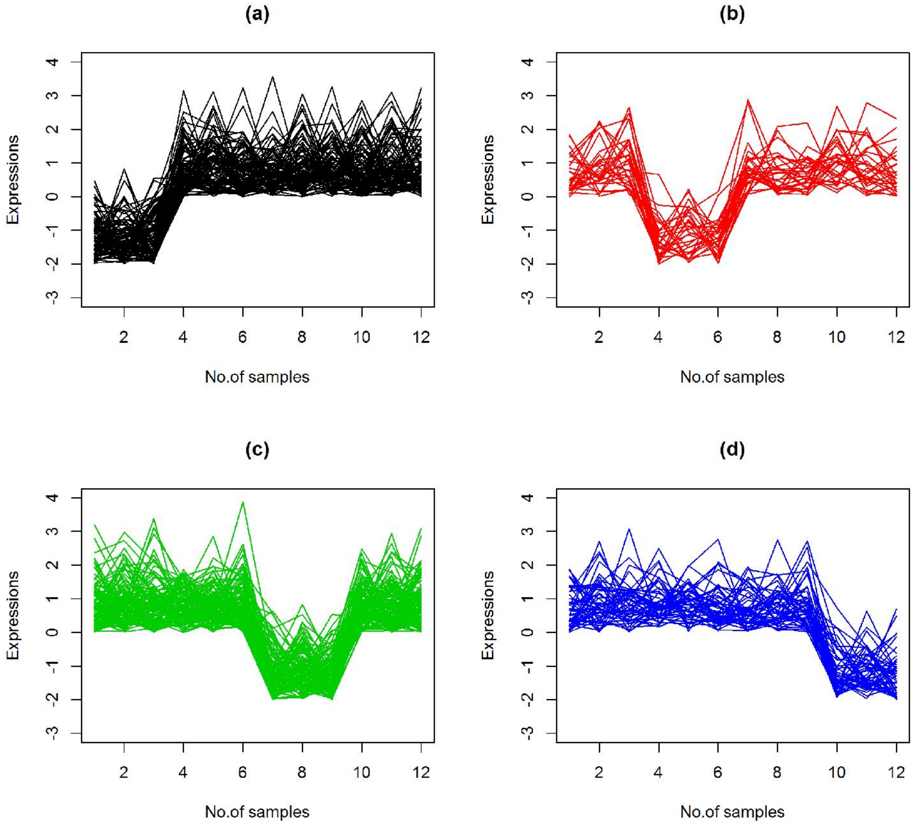

Figure S4. Four different patterns of DE genes for small-sample case (n_1=n_2= n_3=n_4=3). .

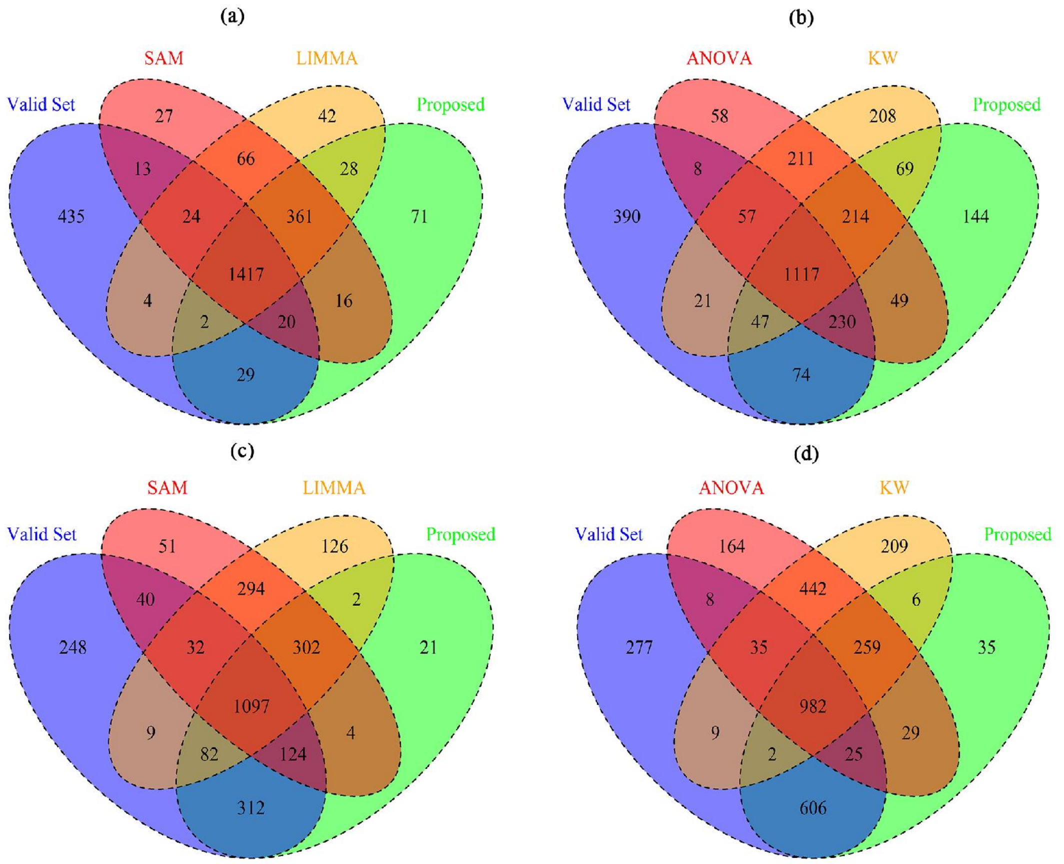

Figure S5. Comparison of the top 1944 selected genes by five methods with 1944 valid DE gene set for Platinum Spike dataset for small-sample case (n_1=n_2=3). In absence of outliers, Venn diagram of top 1944 genes detected by (a) the SAM, LIMMA and Proposed method or by (b) the ANOVA, KW and Proposed method with 1944 valid DE gene set. In presence of one outlier in 20% of 1944 valid DE genes , Venn diagram of top 1944 genes detected by (c) the SAM, LIMMA and Proposed method or by (d) the ANOVA, KW and Proposed method with 1944 valid DE gene set.

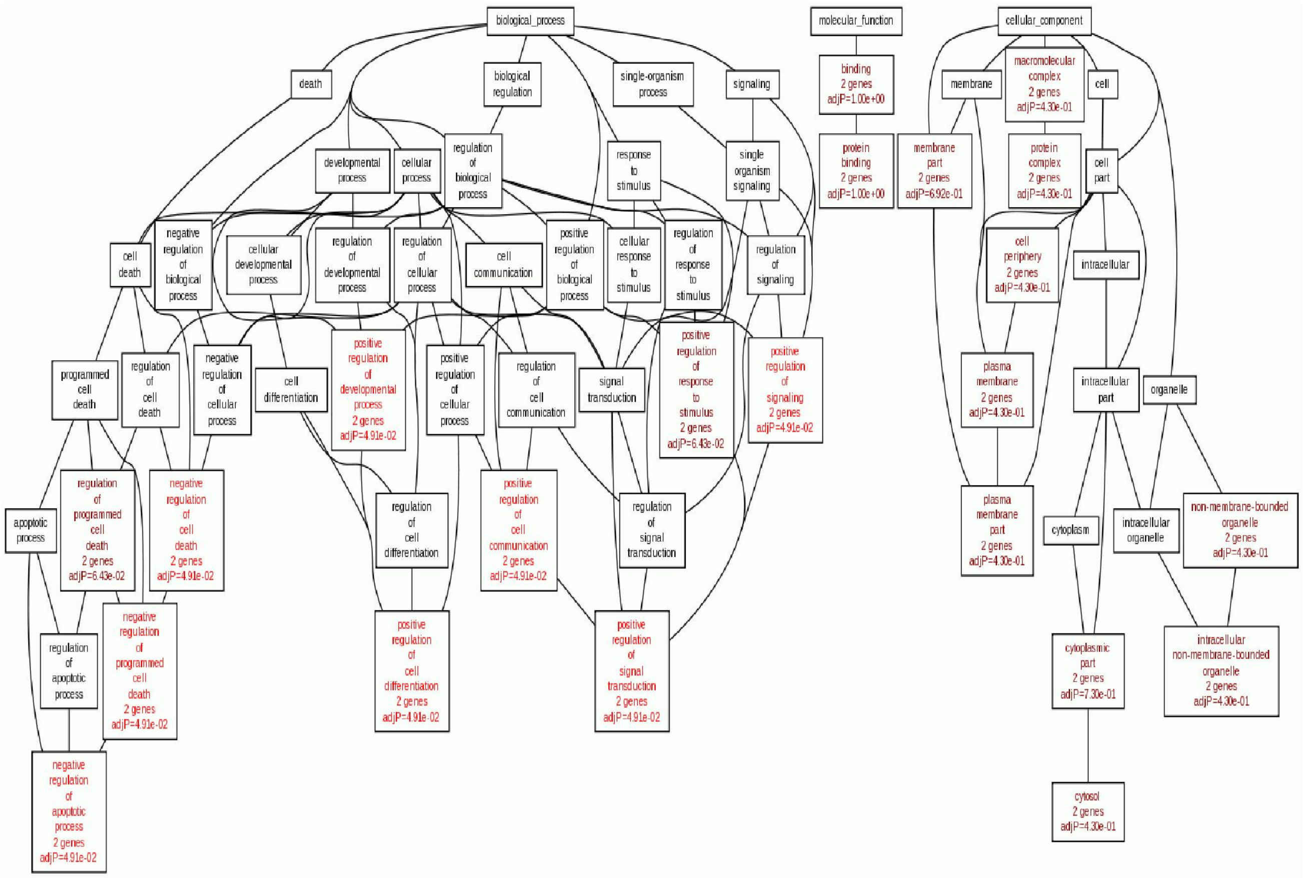

Figure S6. Gene ontology (GO) categories of three (3) genes for BRCA dataset. The directed acyclic graph (DAG) shows the GO categories of three (3) genes, detected by the Proposed method only for breast cancer (BRCA) dataset. In the DAG tree; each box in the tree lists the name of the GO category, the number of genes in the category, and adjusted *p*-value. The box with red categories indicates that they are enriched with adj. *p*-value<0.05.

Table A1. Performance evaluation of different methods based on Spike gene expression dataset.

Methods	In Absence of Outliers				
	TPR	TNR	FPR	FNR	AUC
ANOVA	0.818	0.978	0.021	0.181	0.816
KW	0.809	0.977	0.022	0.190	0.805
SAM	0.833	0.980	0.019	0.166	0.832
LIMMA	0.829	0.980	0.019	0.170	0.828
EB	0.751	0.971	0.028	0.249	0.749
GAGA	0.770	0.973	0.026	0.229	0.768
BRIDGE	0.716	0.967	0.032	0.284	0.713
Proposed	0.833	0.980	0.019	0.167	0.833
Methods	In Presence of Single Outlier in 20% of valid 1944 DE genes				
	TPR	TNR	FPR	FNR	AUC
ANOVA	0.677	0.962	0.037	0.323	0.674
KW	0.673	0.962	0.037	0.326	0.669
SAM	0.736	0.971	0.031	0.261	0.733
LIMMA	0.680	0.963	0.037	0.320	0.678
EB	0.641	0.958	0.041	0.358	0.638
GAGA	0.665	0.961	0.038	0.335	0.662
BRIDGE	0.832	0.982	0.018	0.158	0.832
Proposed	0.833	0.982	0.018	0.157	0.833

In this table the summary statistics (TPR, TNR, FPR, FNR and AUC) are estimated by different methods (ANOVA, KW, SAM, LIMMA, EB, GAGA, BRIDGE and Proposed) based on valid 1944 DE genes for both in absence and presence of one outlier in 20% of valid 1944 genes.
